# Integrative Genomics Reveals Novel Molecular Pathways and Gene Networks for Coronary Artery Disease

**DOI:** 10.1371/journal.pgen.1004502

**Published:** 2014-07-17

**Authors:** Ville-Petteri Mäkinen, Mete Civelek, Qingying Meng, Bin Zhang, Jun Zhu, Candace Levian, Tianxiao Huan, Ayellet V. Segrè, Sujoy Ghosh, Juan Vivar, Majid Nikpay, Alexandre F. R. Stewart, Christopher P. Nelson, Christina Willenborg, Jeanette Erdmann, Stefan Blakenberg, Christopher J. O'Donnell, Winfried März, Reijo Laaksonen, Stephen E. Epstein, Sekar Kathiresan, Svati H. Shah, Stanley L. Hazen, Muredach P. Reilly, the Coronary ARtery DIsease Genome-Wide Replication And Meta-Analysis (CARDIoGRAM) Consortium, Aldons J. Lusis, Nilesh J. Samani, Heribert Schunkert, Thomas Quertermous, Ruth McPherson, Xia Yang, Themistocles L. Assimes

**Affiliations:** 1 Department of Integrative Biology and Physiology, University of California, Los Angeles, Los Angeles, California, United States of America; 2 South Australian Health and Medical Research Institute, Adelaide, South Australia, Australia; 3 School of Molecular and Biomedical Science, University of Adelaide, Adelaide, South Australia, Australia; 4 Department of Medicine/Division of Cardiology, David Geffen School of Medicine, University of California, Los Angeles, Los Angeles, California, United States of America; 5 Department of Genetics and Genomic Sciences, Icahn School of Medicine at Mount Sinai, New York, New York, United States of America; 6 National Heart, Lung, and Blood Institute's Framingham Heart Study, Framingham, Massachusetts, United States of America; 7 Broad Institute of Harvard and MIT, Cambridge, Massachusetts, United States of America; 8 Department of Cardiovascular and Metabolic Research, Biomedical Biotechnology Research Institute, North Carolina Central University, Durham, North Carolina, United States of America; 9 Program in Cardiovascular and Metabolic Disorders and Centre for Computational Biology, Duke-NUS Graduate Medical School, Singapore; 10 Atherogenomics Laboratory, University of Ottawa Heart Institute, Ottawa, Ontario, Canada; 11 John and Jennifer Ruddy Canadian Cardiovascular Research Center, University of Ottawa Heart Institute, Ottawa, Ontario, Canada; 12 Department of Cardiovascular Sciences, University of Leicester, Glenfield Hospital, Leicester, United Kingdom; 13 National Institute for Health Research (NIHR) Leicester Cardiovascular Biomedical Research Unit, Glenfield Hospital, Leicester, United Kingdom; 14 Institut für Integrative und Experimentelle Genomik, University of Lübeck, Lübeck, Germany; 15 DZHK (German Research Centre for Cardiovascular Research), partner site Hamburg, Kiel, Lübeck, Germany; 16 Clinic for General and Interventional Cardiology, University Heart Center Hamburg, Hamburg, Germany; 17 Cardiology Division, Center for Human Genetic Research, Massachusetts General Hospital and Harvard Medical School, Boston, Massachusetts, United States of America; 18 Mannheim Institute of Public Health, Social and Preventive Medicine, Medical Faculty of Mannheim, University of Heidelberg, Mannheim, Germany; 19 Synlab Academy, Mannheim, Germany; 20 Science Center, Tampere University Hospital, Tampere, Finland; 21 Cardiovascular Research Institute, Washington Hospital Center, Washington, D.C., United States of America; 22 Cardiovascular Research Center, Massachusetts General Hospital and Harvard Medical School, Boston, Massachusetts, United States of America; 23 Department of Medicine, Duke University Medical Center, Durham, North Carolina, United States of America; 24 Cleveland Clinic, Cleveland, Ohio, United States of America; 25 Cardiovascular Institute, Perelman School of Medicine at the University of Pennsylvania, Philadelphia, Pennsylvania, United States of America; 26 DZHK (German Research Centre for Cardiovascular Research), partner site Munich Heart Alliance, Munich, Germany; 27 Deutsches Herzzentrum München, Technische Universität München, Munich, Germany; 28 Department of Medicine, Stanford University School of Medicine, Stanford, California, United States of America; University of Wisconsin-Madison, United States of America

## Abstract

The majority of the heritability of coronary artery disease (CAD) remains unexplained, despite recent successes of genome-wide association studies (GWAS) in identifying novel susceptibility loci. Integrating functional genomic data from a variety of sources with a large-scale meta-analysis of CAD GWAS may facilitate the identification of novel biological processes and genes involved in CAD, as well as clarify the causal relationships of established processes. Towards this end, we integrated 14 GWAS from the CARDIoGRAM Consortium and two additional GWAS from the Ottawa Heart Institute (25,491 cases and 66,819 controls) with 1) genetics of gene expression studies of CAD-relevant tissues in humans, 2) metabolic and signaling pathways from public databases, and 3) data-driven, tissue-specific gene networks from a multitude of human and mouse experiments. We not only detected CAD-associated gene networks of lipid metabolism, coagulation, immunity, and additional networks with no clear functional annotation, but also revealed key driver genes for each CAD network based on the topology of the gene regulatory networks. In particular, we found a gene network involved in antigen processing to be strongly associated with CAD. The key driver genes of this network included glyoxalase I (*GLO1*) and peptidylprolyl isomerase I (*PPIL1*), which we verified as regulatory by siRNA experiments in human aortic endothelial cells. Our results suggest genetic influences on a diverse set of both known and novel biological processes that contribute to CAD risk. The key driver genes for these networks highlight potential novel targets for further mechanistic studies and therapeutic interventions.

## Author Summary

Sudden death due to heart attack ranks among the top causes of death in the world, and family studies have shown that genetics has a substantial effect on heart disease risk. Recent studies suggest that multiple genetic factors each with modest effects are necessary for the development of CAD, but the genes and molecular processes involved remain poorly understood. We conducted an integrative genomics study where we used the information of gene-gene interactions to capture groups of genes that are most likely to increase heart disease risk. We not only confirmed the importance of several known CAD risk processes such as the metabolism and transport of cholesterol, immune response, and blood coagulation, but also revealed many novel processes such as neuroprotection, cell cycle, and proteolysis that were not previously implicated in CAD. In particular, we highlight several genes such as *GLO1* with key regulatory roles within these processes not detected by the first wave of genetic analyses. These results highlight the value of integrating population genetic data with diverse resources that functionally annotate the human genome. Such integration facilitates the identification of novel molecular processes involved in the pathogenesis of CAD as well as potential novel targets for the development of efficacious therapeutic interventions.

## Introduction

Coronary artery disease (CAD) remains a leading cause of death worldwide despite a variety of available interventions to reduce cardiovascular events. CAD is partly familial [1], [2], which motivates genetic studies to elucidate novel pharmacological targets. However, large-scale genome-wide association studies (GWAS) have revealed a complex genetic architecture of CAD susceptibility with modest effect sizes for the single nucleotide polymorphisms (SNPs) detected to date [3], [4]. The heritability explained by the top SNPs is approximately 10%, whereas the estimates of total heritability from family studies are substantially higher, between 30% and 50% [1], [2]. Furthermore, the SNP associations themselves rarely provide evidence on their downstream functional consequences, which has prompted the need to integrate DNA variants with functional data to better understand the pathogenic processes.

Genes and their downstream products comprise a complex regulatory machinery that sustains the delicate homeostasis of an organism in a changing environment [5]. Genetic variants can perturb parts of this regulatory network and its ability to restore and maintain homeostasis in the presence of environmental pressure. Consequently, the dysregulated biological processes such as cholesterol metabolism and transport can eventually lead to CAD [6]. To elucidate additional as yet unidentified CAD-related processes, regulatory and functional data on the intermediate tissue-specific molecular phenotypes are essential [7]–[9]. Regulatory networks between genes can be captured by various network reconstruction algorithms [10]–[13]; functional information of genetic variants can be derived from expression quantitative trait loci (eQTLs; contain expression SNPs or eSNPs) that inform on the downstream target genes of genetic variants [8], [14]–[16]. Integration of these empirical data allows us to aggregate eSNPs from multiple interacting genes into eSNP sets that collectively perturb a part of the regulatory network. Subsequently, the eSNP sets can be directly compared with SNP-to-disease associations from a GWAS to connect gene networks to disease.

**Figure 1:**
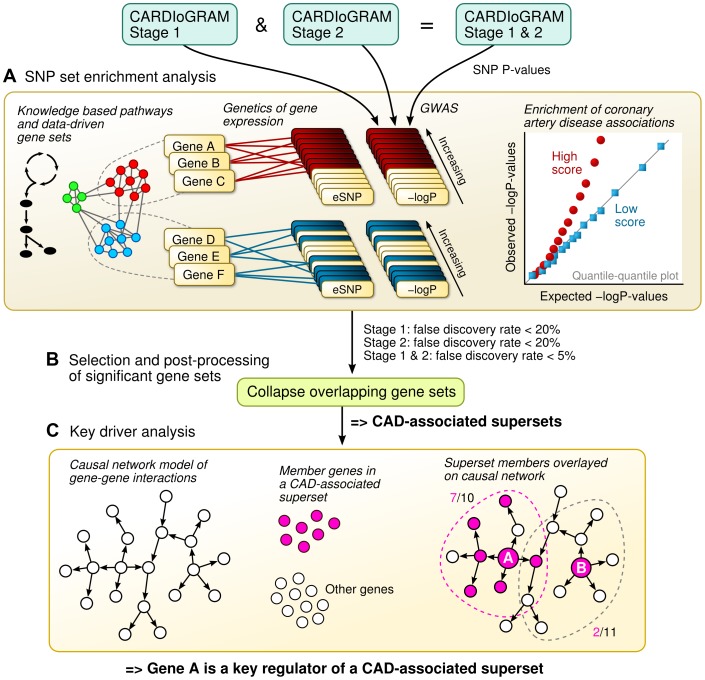
Schematic overview of the study design. A) The SNP set enrichment analysis (SSEA) comprised four steps. First, gene sets from knowledge-driven pathways and data-driven co-expression modules were collected. Second, the gene sets were converted to expression SNP (eSNP) sets according to genetics of gene expression or eQTL studies. Third, P-values from CAD GWAS were extracted for each eSNP. Fourth, the GWAS P-values within eSNP sets were compared against random expectation to derive pathways and network modules enriched for CAD genetic signals. B) Overlapping CAD-associated gene sets were merged and trimmed into non-overlapping supersets. C) Integration of Bayesian gene-gene network models with CAD-associated supersets to determine key driver genes based on network topology.

In this study, we apply an integrative genomics framework (illustrated in Figure 1) to identify the genetically perturbed regulatory networks that contribute to CAD. We make use of four distinct types of data sources. First, associations between SNPs and CAD were determined in 16 independent GWAS – 14 from the CARDIoGRAM Consortium and two from the Ottawa Heart Institute [4], [17]. Second, the effects of SNPs on gene regulation were determined according to eQTLs in multiple tissue-specific genetics of gene expression studies of CAD-related tissues or cell types in humans. As a result, we were able to link the CAD SNPs from the GWAS with their empirically defined target genes. Third, we downloaded known metabolic and signaling pathways (in the form of gene sets) from public repositories, and complemented these known pathways with data-driven network modules of co-expressed genes from multiple transcriptomic studies, to investigate the collective genetic risk via multiple functionally related genes. Finally, we overlaid the identified CAD-associated gene sets onto causal network models of gene-gene interactions from multiple genomic studies to pinpoint the most central regulatory genes. This combination of human genetics, functional genomics, tissue-specific gene networks from empirical data, and biological knowledge in this integrative genomics framework provides us with further insights into the known and hitherto unknown pathogenic processes that are relevant for CAD.

## Results

### SNP set enrichment analysis (SSEA) of canonical pathways

Our first aim was to test if any of the known biological pathways curated in Reactome, Biocarta and Kyoto Encyclopedia of Genes and Genomes (KEGG) databases [18], [19] was more likely to harbor tissue-specific eSNPs that were also associated with CAD in GWAS (Figure 1A). To reduce false discovery and identify the most robust signals, we implemented a multi-stage design that utilized two independent sets of CAD meta-analysis (Stage 1 and Stage 2) each involving distinct sets of CAD cohorts, as well as the overall meta-analysis of all 16 CAD cohorts (combined Stage 1+2 set; see Methods for details). The QQ plots of these three sets of meta-analysis are shown in Figure S1. As majority of the cohorts were from the CARDIoGRAM consortium, the GWAS results from our new meta-analysis closely resembled those of CARDIoGRAM reported previously [4] and there were no new loci identified at genome-wide significance level (Table S1). Tissue-specific eSNPs from CAD related human cells or tissues including adipose tissue, liver, human aortic endothelial cells (HAECs), blood, as well as a pooled eSNP set from multiple tissues and cell types (denoted as ‘All eSNPs’), were used for eSNP-to-gene mapping, yielding five sets of eSNPs mapped to each pathway (Materials and Methods). Each eSNP set representing a pathway was then compared with random eSNP sets drawn from the background eSNPs of matching tissue to look for enrichment of low p value associations with CAD in GWAS using SSEA. Enrichment was measured by a score calculated as the mean -log P-value from Kolmogorov-Smirnov test and Fisher's exact test (see Methods) in SSEA. We defined a pathway to be significantly associated with CAD when permutation-based false discovery rates (FDRs) from Stage 1, Stage 2, and combined Stage 1+2 analyses simultaneously reached 20%, 20%, and 5%. Considering that Stage 1 and Stage 2 GWAS datasets were independent, the combined FDR from these two sets of analysis was expected to be <5% (20% * 20% = 4%). An additional requirement for FDR<5% from the combined Stage 1+2 analysis further ensured low FDR.

To test whether our method can pick up true positive signals, we selected two predefined CAD gene sets based on the GWAS Catalog [20] and CADgene database [21] (details in Methods) as positive controls. These positive controls exhibited strong and consistent signals across multiple sets of SSEA using eSNPs from individual tissues (7<score<29; equivalent to 1e-7<p<1e-29), supporting the sensitivity of our approach. A total of 79 out of 833 canonical pathways tested were associated with CAD in at least one of the five sets of SSEA using different eSNP sets (Table 1; full results in Table S2). Among these, the lipid and lipoprotein pathway from Reactome was significant in adipose eSNP analyses (score 9.8). On the other hand, the bile acid recycling pathway was strongly indicated by the liver eSNPs (score 8.5). The next large group of CAD-associated pathways was related to the immune system: ‘Immunoregulation with lymphoid and non-lymphoid cells’ was the top Reactome pathway (score 11.4 for adipose eSNPs), ‘Antigen processing and presentation’ from KEGG was significant in the liver (score 10.8), and ‘Adhesion and diapedesis of granulocytes’ from Biocarta was a significant pathway when using the HAEC eSNPs (score 3.7). A number of pathways that were directly related to the vascular system or heart, and four pathways that were related to blood coagulation were also associated with CAD. For instance, the SSEA implicated the vascular endothelial growth factor (VEGF), hypoxia and angiogenesis pathway, and the platelet/endothelial cell adhesion molecule 1 (PECAM1) pathway. In essence, the analysis of the curated pathways was able to detect genetic links between CAD and its classical risk factor dyslipidemia, and other suspected CAD processes such as inflammation and vascular dysfunction. More importantly, the use of tissue-specific eSNPs sets helped implicate the most relevant tissues for the significant pathways.

**Table 1:** Knowledge-based grouping of canonical pathways that were significantly enriched for CAD genetic loci.

Category	Selected pathways	All eSNPs	Adipose	Liver	Blood	HAEC
Control	GWAS Catalog	29.0*	9.0*	25.1*	17.2*	7.3*
	CADGene	12.0*	11.9*	9.1*	1.3	1.7
Lipids (9)	Metabolism of lipids and lipoproteins (Reactome)	10.0*	9.8*	2.4	0.7	1.0
	Fatty acid metabolism (KEGG)	5.2*	10.3*	1.6	0.4	0.7
	Recycling of bile acids and salts (Reactome)	5.3*	-	8.5*	-	-
Immune system (24)	Immunoregulation between lymphoid and other cells (Reactome)	9.4*	11.4*	8.6*	1.8	1.6
	Antigen processing and presentation (KEGG)	8.9*	8.1*	10.8*	2.9*	3.0
	Th1/Th2 differentiation (Biocarta)	6.6*	5.1*	5.3*	2.0	0.2
	Adhesion and diapesis of lymphocytes (Biocarta)	3.3	6.0*	-	0.8	3.6*
	Adhesion and diapedesis of granulocytes (Biocarta)	3.3	3.8*	-	0.9	3.7*
Cellular stress response (6)	VEGF, hypoxia and angiogenesis (Biocarta)	4.5*	7.7*	5.9*	3.2	1.4
	Erythropoietin mediated neuroprotection through NF-kB (Biocarta)	3.0*	4.8*	4.6*	2.2	2.6
	Hypoxia-inducible factor in the cardiovascular system (Biocarta)	1.5	3.8*	2.6	1.9	1.1
Cell cycle and growth (18)	Notch-HLH transcription (Reactome)	2.5	3.3*	-	-	0.6
	NRAGE signals death through JNK (Reactome)	3.2*	3.7*	2.5	5.3*	0.5
	EGF signaling pathway (Biocarta)	1.7	2.7*	1.7	1.8	1.2
	G1/S transition (Reactome)	1.2	0.6	5.3*	0.1	0.2
DNA and RNA (7)	Double-strand break repair (Reactome)	3.1*	2.5	2.3	3.3*	0.7
	Spliceosome (KEGG)	1.7	0.6	0.3	5.9*	0.5
Protein metabolism (6)	Metabolism of proteins (Reactome)	2.1*	4.2*	1.1	2.6	1.5
	Proteasome (KEGG)	0.9	0.3	3.6*	0.2	0.7
	Post-translational protein modifications (Reactome)	1.3	3.6*	2.3	0.7	1.5
Other (6)	Bioactive peptide induced signaling (Biocarta)	3.2*	5.9*	0.3	1.5	0.2
	PPAR signaling pathway (KEGG)	3.1*	5.9*	0.6	0.6	0.7
	Glycine, serine and threonine metabolism (KEGG)	2.4*	2.2*	1.3	2.4	3.4

The enrichment score was defined as the mean of negative log-transformed Kolmogorov-Smirnov and Fisher P-values for over-representation of high-ranking GWAS SNPs among the eSNPs that affect the expression of the pathway member genes. The number in parentheses in the first column indicates the number of CAD-associated pathways (detailed in Table S1).

*FDR<20% in Stage 1 and 2 respectively, and FDR<5% in combined Stage 1 & 2.

Our analyses also revealed pathways that so far have not been clearly implicated in CAD development. These included four pathways related to the nervous system (such as ‘Erythropoietin mediated neuroprotection through NF-kB’ and ‘TrkA receptor signaling pathway’ from BioCarta), 13 cell cycle and proliferation pathways (such as ‘NRAGE signals death through JNK’ from Reactome and ‘EGF signaling’ from BioCarta), and ten DNA or RNA pathways (such as ‘Spliceosome’ from KEGG and ‘E2F regulation of DNA replication’ from Reactome). Furthermore, we observed pathways such as ‘Phase I functionalization’ from Reactome and ‘Proteasome’ from KEGG that have a role in the disposal and neutralization of harmful molecules. Pathways that covered amino acids and peroxisome proliferator-activated receptors were also among the significant signals.

We compared the top knowledge-driven pathways detected with our eSNP-based SSEA to those detected by several widely used location-based gene-to-SNP mapping methods for gene set enrichment analysis including iGSEA4GWAS [22], MAGENTA [23] and GSA-SNP [24], and observed considerable variation in the results between methods, with relatively greater consistency between our SSEA approach and iGSEA4GWAS (data not shown). The results from iGSEA4GWAS are reported in a separate manuscript by Ghosh et al (under review). We found that signals from SSEA such as lipid metabolism, immune and inflammatory pathways, PDGF signaling, NOTCH signaling, and PPAR signaling could be replicated in one or more of the other methods tested. Nonetheless, SSEA and iGSEA4GWAS each yielded additional biologically plausible pathways.

Our current analysis included majority of the large-scale CAD-related eQTL sets published before mid 2013. During the revision of this manuscript, several additional blood eQTLs became available [25]–[27]. We tested the pathways identified in our study using the updated blood eQTLs and found minimal impact on our main results, with Pearson correlation coefficient of the pathway scores being 0.96 (comparison between scores before and after incorporating the new blood eQTLs is shown in Table S3).

### SSEA of co-expression modules and formation of non-overlapping supersets

To uncover hitherto unknown biological processes, we augmented the set of canonical pathways with data-driven co-expression modules (empirical sets of tightly co-regulated genes) from multiple previous human and mouse studies (detailed in Materials and Methods). A total of 341 of the 2,706 modules tested satisfied the FDR<20% in both Stage 1 and Stage 2, and FDR<5% in the combined meta-analysis (Table S4). Given that canonical pathways defined by different databases may overlap and also co-expression modules may overlap with known biological pathways, we collected all CAD-associated gene sets regardless of their source (79 canonical pathways + 341 co-expression modules = 420 in total), and analyzed their overlap structure (Figure 1B; overlap matrix in Figure S2). After merging CAD gene sets with overlap of >20% in their member genes (details in Materials and Methods), 62 non-overlapping merged supersets remained.

To ensure the merged supersets still captured the features of the significant pathways, we performed a second round of SSEA on the supersets and applied a stringent Bonferroni-corrected statistical cutoff (correcting for the total number of pre-merged gene sets, n = 3539, not 62 supersets) to focus on the most reliable signals. Therefore, although the second round of SSEA was mainly confirmatory to ensure that we did not lose the signals during merging/trimming, a highly stringent Bonferroni-correction that considered multiple testing of 3539 original gene sets (not 62 supersets after merging) further ensures that the signals passed the threshold were truly robust. Note that this level of Bonferroni correction is highly conservative because we are treating all 3539 gene sets as independent. In reality, the highly overlapping structures among these gene sets make the number of truly independent gene sets much smaller.

Out of the 62 non-overlapping supersets, 22 were confirmed to be genetically associated with CAD in the second round of SSEA (Table S5) and the top six supersets are summarized in Table 2. The data-driven supersets implicated lipid metabolism (‘Lipid I’ and ‘Lipid II’), the immune system (‘Immunity’ and ‘Antigen’) and coagulation processes (‘Lipid II’), consistent with the findings from the canonical pathways. Eight supersets did not significantly overlap with any known pathway or process and could not therefore be annotated by functional categories (hence named “Unknown”; Table S5).

**Table 2:** CAD enrichment scores for selected non-overlapping supersets after the merging of CAD-associated canonical pathways and co-expression modules.

Superset	Overlap with known processes	All eSNPs	Adipose	Liver	Blood	HAEC
Lipid I	Lipid, fatty acid and steroid metabolism; oxidoreductase; PPAR signaling; mitochondrial beta-oxidation; branched-chain amino acid degradation; cholesterol biosynthesis; unsaturated fatty acid biosynthesis	5.4*	9.4*	0.4	0.4	0.7
Lipid II	Lipid, fatty acid and steroid metabolism; oxidoreductase; vesicles; xenobiotics; complement and coagulation system	10.3*	11.0*	1.9	1.8	0.1
Antigen	Human leukocyte antigens; bone reabsorption	10.3*	9.5*	8.6*	3.7	1.1
Immunity	Wound and inflammatory responses; cell activation	6.1*	7.4*	8.7*	1.5	1.4
Unknown I	-	3.5	7.4*	4.4	1.2	0.2
Unknown II	-	2.9	6.4*	3.9	2.2	0.2

The enrichment score was defined as the mean of negative log-transformed Kolmogorov-Smirnov and Fisher P-values for over-representation of high-ranking GWAS SNPs among the eSNPs that affect the expression of the superset member genes.

*P<0.05 in either Fisher's exact test or Kolmogorov-Smirnov test after Bonferroni correction for the 3,539 original gene sets.

### Key driver analysis (KDA) of CAD-associated gene supersets

In order to determine the regulatory genes (referred to as key drivers) at the center of the CAD-associated supersets as a means to further explore regulatory mechanisms and prioritize disease genes, we performed KDA using tissue-specific Bayesian network models constructed from transcriptomic and genetic datasets from multiple human and mouse studies (Figure 1C; details in Materials and Methods) [12], [28], [29]. The topology of these Bayesian networks captures detailed gene-gene regulatory relationships and can help infer key network drivers. The KDA results for the top six supersets are summarized in Table 3 and full list of key drivers are in Table S6. To test if the key driver genes were also responsible for the enrichment of CAD genetic signals in each of the CAD superset, we also ranked and selected the member genes whose eSNPs (i.e., SNPs that are associated with the expression levels of the member genes) showed the strongest CAD association within the superset, termed “GWAS signal genes”, for comparison (Table 3). Interestingly, the key driver genes were mostly different from the GWAS signal genes, which supports a previously observed phenomenon [30] that important regulatory genes may not harbor common susceptibility polymorphisms by natural selection and, conversely, that a majority of common disease susceptibility loci (as captured by GWAS) do not involve key regulatory genes but are situated in the periphery of biological networks.

**Table 3:** Top five genes whose eSNPs show strongest association with CAD in GWAS (termed “GWAS signal genes”) and key driver genes for selected CAD-associated supersets.

Superset	GWAS signal genes*	Key driver genes#
Lipid I	*SREBF1, LPL, LDLR, CYP4A11, ME1*	*DCI, SQLE, ETHDH, SLC22A5, EHHADH*
Lipid II	*TMEM116, TMEM27, MAT1A, LRRC19, NAT2*	*GC, CES3, PZP, HGR, PLG*
Immunity	*CTSS, HLA-B, OAS1, HLA-DRB1, HLA-DQB1*	*PTPRC, NCKAP1L, FCGR1A, FYB, FCER1G*
Antigen	*CD2AP, AS3MT, HCG4, TAF11, FLOT1*	*VPS52, PPIL1, GLO1, GFER, DECR2*
Unknown I	*NT5C2, SURF6, ARL3, LMO4, TIE1*	*DNAJC7, UBE2S, ALG8, ZC3H7B, PRMT1*
Unknown II	*ALS2CR13, TMEM116, C10orf26, CEACAM3, NM_152451*	*CEBPD, SGK1, SLC10A6, KCNA5, MAP3K6*

^*^Genes within superset whose eSNPs (i.e. putative functional SNPs that affect gene expression) show best association with CAD in the GWAS meta-analysis.

# The key driver genes were ascertained by combining key driver analyses of all available Bayesian networks, and taking into account both the consistency across datasets and the KDA statistics.

**Figure 2:**
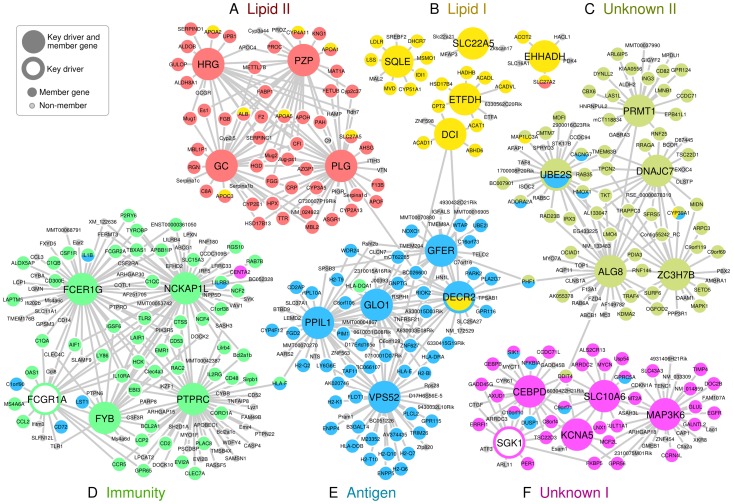
Key driver genes of six CAD-associated supersets, and their adjacent regulatory partners. Key driver genes were denoted as larger nodes in the network. Genes were colored based on their membership in the six CAD-associated supersets. A) ‘Lipid II’ superset in red. B) ‘Lipid I’ superset in yellow. C) ‘Unknow II’ superset in lime. D) ‘Immunity’ superset in green. E) ‘Antigen’ superset in blue. F) ‘Unknown I’ superset in magenta. Only edges that were present in at least two Bayesian networks constructed from independent studies were included.

Both the ‘Lipid I’ and ‘Lipid II’ supersets fell under the general category of lipid and fatty acid metabolism (Figure 2A and 2B). They share 14% of their members, including seven apolipoproteins, but were considered non-overlapping according to our *a priori* overlap threshold of 20%. In fact, the key drivers for ‘Lipid I’ comprise genes important for fatty acid metabolism (*DCI*, *ETFDH* and *EHHADH*) and cholesterol biosynthesis (*SQLE*), whereas ‘Lipid II’ was regulated by coagulation (*PLG* and *HRG*) and carrier proteins (*GC* and *PZP*), which confirms non-overlapping functionality between the two supersets. Of note, two critical genes involved in lipoprotein metabolism, *LPL* and *LDLR*, were among the top GWAS signals genes for ‘Lipid I’.

Two supersets of immune system genes – ‘Antigen’ and ‘Immunity’ - were significantly enriched for CAD loci in adipose tissue and the liver (Figure 2D and 2E). The ‘Antigen’ superset owes its annotation to the human leukocyte antigen (*HLA*) and mouse *HLA* orthologs (*H2* genes) that comprise 21 of the 221 member genes. We found the key drivers for this superset such as *GLO1*, *PPIL1* and *DECR2* to be highly consistent across Bayesian networks from multiple tissues (Table S7). The ‘Immunity’ superset contains a variety of immune response genes including six *HLA* genes and 18 cytokines or their receptors such as *CCL2*, CD antigen genes, *CXCL10*, *IL2RB* and *TLR2*. Four of the top five key drivers for the ‘Immunity’ superset (*PTPRC*, *FYB*, *FCGR1A* and *FCER1G*) participate in the immune response, and three (*PTPRC*, *FYB* and *FCER1G*) have been previously identified as key drivers of an inflammatory gene signature underlying multiple diseases (including CAD) [12].

We could not annotate two out of our six top supersets (‘Unknown I’ and ‘Unknown II’). These supersets, however, have consistent network key drivers across multiple tissues (Figure 2C and 2F, Table S7). ‘Unknown I’ contained a diverse set of key driver genes such as *SGK1*, *SIK1* and *SLC10A6* (sodium metabolism and hypertension), *MT2A* and *TSC22D3* (glucocorticoid signaling), *GADD45G*, *ERRFI1*, *GPRC5A*, and *EGFR* (cell growth and apoptosis), and *CEBPB*, *CEBPD*, and *KCNA5* (heart development and function). Possible functions of ‘Unknown II’ include RNA metabolism (*ZC3H7B*), protein methylation (*PRMT1*), glycosylation (*ALG8*), chaperone recycling (*DNAJC7*) and ubiquitination (*UBE2S*), and similar annotations could also be found for the network neighboring genes of these key drivers. Of note, the gene *CYP39A1* which converts cholesterol into bile acid was shared between ‘Lipid I’, ‘Antigen’ and ‘Unknown II’ supersets.

### Experimental validation of the key drivers of the top-scoring superset

The ‘Antigen’ superset had the highest combined CAD association score across the five sets of SSEA using eSNP sets from different tissues (Table 2) and their key driver genes identified were highly consistent across the Bayesian networks used for KDA (Table S7). For these reasons, we tested the effects of silencing three of the key drivers, glyoxalase I (*GLO1*), peptidylprolyl isomerase I (*PPIL1*) and peroxisomal 2,4-dienoyl CoA reductase 2 (*DECR2*), on the expression of member genes in the ‘Antigen’ superset in HAECs, aiming to validate the role of these key drivers in regulating this CAD superset. HAECs were chosen based on their critical role in maintaining a healthy vessel wall and knowledge that endothelial dysfunction is observed early in the development of atherosclerosis [31]. Of note, the number of HAEC specific eSNPs was relatively low due to the limited sample size in the original study [32], which could explain the lack of significant pathway enrichment signals in this cell type in this study. However, this statistical power issue should not be misconstrued as a lack of relevance of this cell type in the pathogenesis of CAD.

Three separate siRNAs against the *GLO1* transcript NM_006708 (Qiagen Catalog Numbers SI04175892, SI04206244, SI04266052) resulted in 86%, 88% and 91% reduction in *GLO1* expression, and siRNA SI04284224 against the *PPIL1* transcript NM_016059 resulted in 87% reduction. *DECR2* expression level was too low in HAECs to be informative. Whole genome transcript expression in response to *GLO1* and *PPIL1* suppression was measured using microarrays and then compared with that from scrambled siRNAs (null control) to detect genes with significant changes.

A total of 485 and 656 genes were affected by *GLO1* and *PPIL1* suppression, respectively (P<0.001 for both). Due to substantial overlap (281 genes were affected by both *GLO1* and *PPIL1*), we pooled the 860 unique genes that were significantly affected by either *GLO1* or *PPIL1*. We determined how many of these top genes were neighbors by two edges to any of the five key drivers (*GLO1*, *PPIL1*, *DECR2*, *VPS52* and *GFER*) of the ‘Antigen’ superset when considering all available Bayesian networks. We found the 547 neighbor genes to be enriched for the differentially expressed genes by 1.7 fold (37 observed vs. 21.5 expected, P = 5.5×10^−4^ by Fisher's exact test), indicating that the suppression of *GLO1* and *PPIL1* perturbed the ‘Antigen’ superset in HAEC.

To verify the connection of *GLO1* and *PPIL1* perturbations to CAD, we tested the two pre-defined CAD positive control gene sets (Table 1) against the differential P-values from the siRNA experiments. The CADgene positive control set was significantly associated with altered expression due to either *GLO1* (P_Fisher_<0.0001, P_K-S_ = 0.0042) or *PPIL1* suppression (P_Fisher_<0.0001, P_K-S_ = 0.0085). These results indicate that the expression levels of genes from CAD-related processes are significantly more affected by *GLO1* and *PPIL1* knockdown compared to a random set of genes in HAEC.

## Discussion

We performed an integrative genomics study that combined association signals from a large GWAS, tissue-specific eQTL datasets, known canonical pathways, and data-driven regulatory networks to gain insights into the causal molecular mechanisms of CAD. Our approach identified both established and novel biological processes supported by functional evidence; specifically, the expression levels of the member genes within these processes were controlled by multiple CAD-associated SNPs. To dissect the key regulatory mechanisms, we derived a network representation of the central genes involved in the pathogenic processes and investigated how the affected genes were related to known biological pathways and metabolic cascades and how the processes were inter-connected in multi-tissue regulatory networks. Our study revealed a highly complex and multifactorial genetic basis for CAD, and implicated several known and novel causal pathways along with their potential regulators deserving of further study.

Several aspects of this study distinguish it from previous pathway and network studies of CAD. First, we used data from eQTL studies of CAD-related tissues or primary cell types to assign eSNPs to genes, whereas previous approaches have primarily utilized genomic location for assignment of SNPs to genes [3], [22], [23], [33], [34]. Our method incorporates empirical functional support and tissue specificity into the analyses to increase the sensitivity of detecting tissue-specific molecular events that would have been missed by conventional methods [22], [23], [33] and to enhance the biological and mechanistic interpretability of the disease-related signals [9], [35]–[37]. Our partial re-analysis of data incorporating recently reported blood eSNPs suggests that the addition of new eQTLs reinforces the significance of the pathways identified thus far. Second, we tested both knowledge-driven and data-driven gene sets to expand the coverage of novel biological processes. Third, we used two large independent CAD GWAS meta-analyses, merged and trimmed overlapping CAD-associated gene sets, and imposed a strict Bonferroni threshold for final statistical evaluation of the CAD signals to avoid false positives and focus on the most reliable core processes for CAD. Fourth, we utilized scores of empirically-derived gene networks from diverse CAD-related tissues to extract the CAD network architecture and the key driver genes, whereas previous studies have relied on literature-based topologies, protein-protein interaction networks, or single-tissue networks [3], [38]–[40]. Lastly, we performed targeted siRNA studies in HAEC to provide experimental support for our *in silico* findings.

Of note, the KDA approach we utilized has been recently demonstrated by multiple studies to have the capacity to identify hidden novel regulatory genes that are missed by traditional analysis, and novel predictions from each of these studies have been experimentally validated [12], [28], [41]. The key drivers identified, however, are not necessarily GWAS hits. In contrast, it is their downstream or peripheral genes that are more likely to be identified in GWAS and the expression of these genes are more likely to be *cis*-regulated by GWAS SNPs. This may explain why a majority of the GWAS hits uncovered to date only have small effects on complex disease phenotype. As elucidated previously by Goh et al. [30], the lack of GWAS signals from key driver genes can be explained by evolutionary constraints imposed on important regulators because strong genetic perturbations in these key regulators are more likely to be deleterious. If certain genetic polymorphisms within key regulatory genes (e.g., transcription factors) indeed successfully segregate in general population and can be identified in GWAS, these polymorphisms tend to be *cis*-eSNPs of the regulators themselves and then *trans*-eSNPs of additional disease genes [25].

Our results support the role of genetic perturbation to lipid metabolism, immune response and inflammation, coagulation, and vascular wall function in the etiology of CAD. Apart from cholesterol metabolism and transport, a causal role for many of these processes in pathogenesis of CAD has been debated for years. For instance, recent Mendelian randomization studies as well as randomized control trials of cardiovascular drugs have demonstrated that a number of known key genes within these pathways (e.g. CRP, fibrinogens) are not causally associated with CAD [42]–[44]. Our results suggest that a critical mass of causal variants may be inherited within many of the genes in these pathways even if the pathway includes some genes that have no causal role in the pathogenesis of CAD.

We identified novel biological processes such as neuroprotection, cell cycle and proteolysis that were perturbed by the CAD-associated genetic variants. Furthermore, the data-driven network models identified CAD-associated gene sets that did not overlap with any known biological processes. We merged the knowledge-based biological pathways and data-driven functional units of genes derived from expression patterns to bridge the knowledge gaps, and focused on six gene sets that were not only strongly associated with CAD in human GWAS but also exhibited a consistent causal network topology around a limited number of key regulatory genes.

The supersets ‘Lipid I’ and ‘Lipid II’ are involved in cholesterol and lipid biosynthesis or degradation. The ‘Lipid II’ superset appears to have more diversified functions beyond lipid biosynthesis and transport, as it contains multiple additional genes from coagulation and complement pathways. If ‘Lipid I’ and ‘Lipid II’ were simultaneously perturbed, one can speculate that the lipid transport system would become overwhelmed, wound healing processes and the complement cascade would become over-activated, and the lipid-rich debris would feed the accumulation of plaque on the vessel wall. Furthermore, two of the central genes in Lipid II, plasminogen (*PLG*) and the histidine-rich glycoprotein (*HRG*) regulate the fine balance between clotting and fibrinolysis, which can affect the propensity of thrombosis after plaque rupture.

The strongest overall CAD association was observed for the ‘Antigen’ superset. The key drivers were highly consistent across tissues but, surprisingly, none of them have been directly implicated in antigen processing. In fact, many of the network driver genes appear to be involved in protein processing, and endosomal and lysosomal functions. For instance, glyoxalase 1 (*GLO1*) plays a critical part in the enzymatic defense against dysfunctional glycated forms of proteins [45], the vacuolar protein sorting 52 homolog (*VPS52*) is involved in the transport and sorting of proteins from the plasma membrane to the lysosome via mannose-6-phosphate receptors [46], the N-acetylglucosamine-1-phosphate transferase gamma subunit (*GNPTG*, top 10 key driver, between *GLO1* and *GFER* in Figure 2) is part of mannose-6-phosphate synthesis [47], and peptidylprolyl isomerase (*PPIL1*) is a member in the cyclophilin family that regulates protein folding and immune responses [48]. Of note, there may also be a direct link to lipid metabolism: the peroxisomal 2,4-dienoyl CoA reductase (*DECR2*), which participates in the beta-oxidation of unsaturated fatty acids [49], is a key driver of this ‘Antigen’ superset and a member of ‘Lipid I’.

*GLO1* is an interesting candidate for a causal CAD gene. Diabetes, kidney disease, and diabetic kidney disease in particular increase the risk and severity of CAD dramatically [50], [51], and the glyoxalase system is an important protective mechanism against the formation and subsequent accumulation of advanced glycation end products that are believed to promote diabetic end-organ damage. In a mouse study, a *Glo1* knock-down model spontaneously developed kidney disease even without diabetes [52], and a single case of human *GLO1* deficiency exhibited both end-stage renal disease and severe atherosclerosis [53]. A recent study demonstrated a protective role of *Glo1* in restoring neovascularization of ischemic tissue in diabetic rats [54]. In our study, knock-down of *GLO1* in HAECs perturbed the expression of many of the same genes that were affected by CAD-associated SNPs in the human GWAS. Therefore, the glyoxalase system may represent one common pathway responsible for both the microvascular and macrovascular complications observed in subjects with diabetes.

The ‘Immunity’ superset may represent the same core inflammatory signature that we have observed across species, tissues, and multiple diseases [12]. Four of the top key drivers (*HCK*, *TYROBP*, *NCKAP1L* and *AIF1*) identified from the previous multi-disease inflammatory signature were also detected as key drivers for the ‘Immunity’ superset in this study (Figure 2D). Although we cannot provide definitive answers on the sequence of events, one may hypothesize that the ‘Immunity’ superset executes the downstream machinery that is recruited in response to the antigen presentation in cells under metabolic stress.

The central genes in the ‘Unknown I’ superset include the serum glucocorticoid regulated kinase 1 (*SGK1*), a mitogen-activated protein kinase (*MAP3K6*), a sodium/bile acid co-transporter (*SLC10A6*) and a C/EBP transcription factor (*CEBPD*). Of these, *SGK1* has been studied the most and is believed to be important for renal sodium absorption, salt-sensitivity to hypertension and glycemia, cardiac repolarization, and numerous other processes [55]. *MAP3K6* regulates *VEGF* expression [56], and its expression was altered in a mouse model of cardiomyopathy [57]. The *SLC10* gene family contains three sodium-dependent transporters, of which *SLC10A6* transports sulfoconjugated steroid hormones. One of the shared genes between ‘Antigen’ and ‘Unknown I’ in Figure 2, salt-inducible kinase 1 (*SIK1*), is thought to be part of the sodium-sensing network [58]. Cyclin-dependent kinase inhibitors (*CDKN*) may be causally related to atherosclerosis [3], [59], although the exact role of *CDNK1A* (adjacent to *MAP3K6* and *SLC10A6* in Figure 2) remains unclear. Thus, the superset ‘Unknown I’ appears bring together processes quite relevant to CAD including glucocorticoid signaling, vascular stress response, cell growth, and blood pressure control.

‘Unknown II’ could not be annotated by known processes and the functions of the core genes remain poorly understood. There were a number of genes that may regulate methylation, histones, chromatin, and splicing (*ING3*, *CBX6*, *LMNB1*, *SFRS5*), post-translational protein modifications and activation (*PRMT1*, *ALG8*, *PDIA3*, *DNAJC7*), ubiquitination (*UBE2S*, *RNF25*, *RNF146*), cytoskeleton organization (*ARPC3*, *MAP1LC3A*, *DYNLL2*, *ARL6IP5*), and cell cycle (*CD82*, *ING3*, *UBE2S*, *MDFI*, *TRAF4*). It is possible that the genes within ‘Unknown II’ participate in the stress-induced epigenetic and proteomic changes that contribute to atherogenic processes. If this is true, it may explain why the curated pathways, many of which consist of chemical reactions between metabolites, missed the predominantly regulatory gene networks such as ‘Unknown I’ and ‘Unknown II’.

We had a wealth of data sources at our disposal in this study to derive a comprehensive view of the complex mechanisms of CAD. Nevertheless, we acknowledge the following limitations. First, our study cannot distinguish pathways or gene subnetworks that are more relevant to specific subtypes of CAD from those which cause CAD through more general mechanisms involving relatively well-understood cardiometabolic processes. Future studies involving sample sets that include more refined subtyping of cases may help further advance our understanding in this respect. Second, although the concept of eQTL as an empirical alternative to the traditional location-based gene-SNP mapping in pathway analysis is appealing from a biological perspective as it carries functional implications and allows detection of tissue-specific signals, in practice, however, the lack of comprehensive and large enough genetics of gene expression studies may limit the power and the biological coverage of the approach, as the total number of eSNPs is typically lower and eSNPs from additional CAD-related cell types of tissues are not necessarily available. On the other hand, emerging resources such as ENCODE [60], [61] and GTEx [62] are likely to improve the situation in the future.

In conclusion, we used an integrative genomics framework to shed light on the key genes and regulatory processes involved in the pathogenesis of CAD. We detected genetically driven perturbations of several pathways with a strong *a priori* evidence of involvement in CAD (cholesterol synthesis, inflammation, and blood coagulation), as well as novel processes (neuroprotection, epigenetic and post-translational modifications, intracellular transport, proteolysis, and cell cycle). The data suggest that many genes in these biological processes are causally associated with CAD even if this may not be the case for all the pathway or network members. We verified the importance of the key drivers in the top-scoring gene set using an experimental gene expression model. Thus, the CAD associated gene networks and key drivers identified in this study warrant further validation in additional population genetic and mechanistic studies. Further knowledge gained through such studies has the potential to lead to major advances in the development of therapeutic strategies to reduce the risk of CAD.

## Materials and Methods

### Overall analysis flow

The overall integrative framework is depicted in Figure 1. First, we applied a modified SNP set enrichment analysis (SSEA) [36], [37] to find sets of functionally related genes that were associated with CAD (Figure 1A). In this analysis, we used knowledge-based canonical pathways and data-driven co-expression network modules as the functional units of genes that were tested for CAD association, tissue-specific eQTL studies to connect the genes to SNPs, and CAD GWAS to provide the associations between SNPs and CAD. To reduce false discovery and identify the most robust signals, we implemented a multi-stage design that leveraged two independent GWAS meta-analyses of CAD. Next, we investigated the statistically significant CAD-associated pathways and co-expression modules for shared genes, and merged any overlapping gene sets into non-overlapping supersets (Figure 1B). Lastly, the key regulator genes for each superset were determined by integrating multiple tissue-specific Bayesian causal network models of gene interactions with the CAD-associated gene supersets (Figure 1C).

### Genome-wide association studies of coronary artery disease

The design, clinical classification and genotyping within the Coronary ARtery DIsease Genome-wide Replication And Meta-Analysis (CARDIoGRAM) Consortium have been described previously [4], [63]. The dataset used in this study comprised 25,491 cases with coronary artery disease, myocardial infarction or both and 66,819 controls from the 14 cohorts within CARDIoGRAM and two GWAS by the Ottawa Heart Institute in collaboration with Cleveland Clinic and Duke University [17].

The 16 GWAS were split into two independent sets (Table S8): The Stage 1 set combined the results from the Ottawa Heart Genomics Study with the Cleveland Clinic Gene Bank (OHGS_A and OHGS_CCGB_B), the CAD component of the Wellcome Trust Case Control Consortium (WTCCC), the Duke CATHGEN Study, and the German Myocardial Infarction Family Studies I, II, III with Collaborative Health Research in the Region of Augsburg (GerMIFS1, GerMIFS2, GerMIFS3/KORA). The remaining CARDIoGRAM cohorts formed the Stage 2 set and included Atherosclerotic Disease VAscular functioN and genetic Epidemiology Study (ADVANCE), CADomics, Cohorts for Heart and Aging Research in Genomic Epidemiology (CHARGE), deCODE CAD, Ludwigshafen Risk and Cardiovascular Health Study (LURIC/AtheroRemo 1, LURIC/AtheroRemo 2), MedStar, Myocardial Infarction Genetics Consortium (MIGen) and the PennCath Study.

Genotyping of the individuals was performed by Affymetrix or Illumina platforms and imputed to 2.5 million SNPs prior to meta-analyses [4]. Ancestry was restricted to European origin by self-reporting or principal component analysis of genotypes or both. The Ottawa cohorts were imputed separately using IMPUTE2 and MACH software, and a reference panel that included 112 European genomes from the 1000 Genomes Project (August 2009) and 298 additional subjects from a separate CEU/TSI reference panel [17].

Correcting of population stratification was performed as described previously [17]. The smartPCA (principal components analysis) program from EIGENSOFT v3.0 [64] was used to identify and remove subjects of admixed or non-European ancestry. Study subjects were processed with 270 HapMap2 subjects for PCA (90 CEU, 90 JPT+CHB, 90 YRI). In the resulting first 2 dimensions from PCA, k-means was used to ascertain the center of each of the CEU, JPT+CHB, and YRI clusters, and the original 2 PC dimensions were projected onto these axes. Subjects were removed if they fell outside an oval whose major axes were 10 times the standard deviation of the CEU cluster along the 2 transformed axes.

The SNP-level associations were estimated by a meta-analysis approach similar to that used for CARDIoGRAM [4]. SNPs with minor allele frequency below 1%, significant Hardy-Weinberg equilibrium (P<0.0001), imputation quality below 50% or call rate below 75% were excluded. Rare SNPs that were present in less than three Stage 1 GWAS or less than five Stage 2 GWAS were also excluded. The GWAS were analyzed jointly by a fixed-effect inverse-variance weighted model within the Stage 1 and Stage 2 sets, respectively. Heterogeneous SNPs with significant Q and I statistics (P<0.0001) were analyzed by DerSimonian and Laird inverse variance model of random effects. We also used all available cohorts to create an overall meta-cohort (denoted as Stage 1+2).

### Knowledge-driven pathways

We included curated pathways from the Reactome, Biocarta and Kyoto Encyclopedia of Genes and Genomes (KEGG) databases [18], [19]. The Reactome database is based on reactions between diverse molecular species rather than limiting the pathways to protein-protein interactions or other types of non-biological categories. Also, the nested structure of the Reactome database helps to increase the coverage to multiple levels of gene set organization. The KEGG database represents carefully curated and experimentally validated pathways of metabolic processes and gene sets of human diseases, while BioCarta is a community based effort to describe interactions that arise from proteomic and other similar studies. In total, 833 gene sets were included in the analyses, collectively referred to as knowledge-driven pathways.

We constructed two positive control gene sets using previously known CAD candidate genes. The first positive control gene set was based on the GWAS Catalog [20]. SNPs with P<5.0×10^−8^ for the traits ‘Coronary heart disease’, ‘Coronary artery calcification’ and ‘Myocardial infarction’ were collected from the catalog, and the reported genes for these loci were included in the control set. Another positive control was formed from the CAD candidate genes curated in the CADgene database [21].

### Data-driven modules of co-expressed genes

Consistent expression patterns among specific sets of genes were investigated in previous studies to define co-regulated gene sets, commonly referred to as co-expression network modules. These modules can be considered data-driven “pathways” of gene regulation that typically operate upstream of the classical pathways of chemical signaling and enzymatic action. We utilized co-expression modules constructed using the weighted Gene Co-expression Network Analysis [13] from ∼10 human and mouse studies that involved multiple CAD-related tissues (details and references in Table S9). Human modules were obtained from HAEC, adipose tissue, blood, and liver. Mouse modules were obtained from adipose tissue, liver, muscle, brain, heart, islet cells and kidney. A total of 2706 co-expression modules representing data-defined functional units of genes were used in this study.

### Expression SNPs (eSNPs) from human expression quantitative trait loci (eQTL) studies

Human eQTL studies are analogous to GWAS of quantitative traits, except that the traits are tissue-specific gene expression levels rather than biomarkers or clinical measures. eQTL studies constitute an important source of empirically supported mappings from a genetic variant (eSNP) to its gene target and these mappings can be reversed to convert a gene set back into the respective eSNP set for direct testing of disease associations in GWAS. The human eSNP data used in this study were collected previously from adipose tissue, liver, HAEC, blood, fibroblasts, lymphoblasts, and monocytes (details and references in Table S9). Both *cis*-eSNPs (within 1 Mb distance from gene region) and *trans*-eSNPs (beyond 1 Mb from gene region) at false discover rate <10% were included. We chose adipose tissue, liver, blood and HAEC for tissue-specific analyses due to their direct relevance to CAD and relative abundance of eSNPs, while all eSNP resources were pooled into a tissue-independent set denoted as ‘All eSNPs’, yielding five sets of gene-eSNP mapping for each gene set.

We observed a high degree of linkage disequilibrium (LD) between eSNPs. If left uncorrected, redundant eSNPs can inflate the disease association scores of a pathway or gene network and increase the number of false positives. For this reason, we devised an algorithm to remove eSNPs in LD while preferentially keeping those with a strong statistical association with gene expression (Text S1). The preferential treatment based on these expression association P-values was motivated by previous observations that disease loci are enriched among eSNPs [36], [65].

We used eSNPs from multiple human cohorts with different sample sizes and study designs. Simply comparing the raw expression association P-values in the LD pruning algorithm could potentially skew the selection according to the study characteristics rather than biological relevance. Therefore, we ranked the expression association P-values and scaled the ranks to the interval between 0 and 1 within the study-specific eSNP dataset before pooling the studies and applying the LD pruning algorithm.

The reduction in the number of accepted eSNPs after LD pruning was smooth over a wide range of LD thresholds (Figure S3). We chose a moderate LD cutoff (R^2^<0.7) that lead to the rejection of approximately 50% of eSNPs. This cutoff was chosen because it preserves statistical power while removing eSNPs in high LD. We used the LD structure of the CEU HapMap population [66] for the eSNP pruning given our CAD GWAS included only subjects of white/European descent.

### SNP set enrichment analysis (SSEA)

We applied a modified SSEA to identify gene sets associated with CAD [36], [37] (Figure 1A). We collected gene sets from knowledge-based pathway databases, or defined them according to data-driven co-expression network modules (Figure 1A, left). We also determined the specific sets of eSNPs that perturb the expression of the member genes in each gene set based on the tissue-specific eSNP sets described above (Figure 1A, middle). We retrieved the CAD association P-values for the eSNPs from the CAD GWAS (Stage 1, Stage 2, Stage 1+2, separately), compared the P-values against the random expectation, and summarized the observed difference as a single enrichment score, as detailed below (Figure 1A, right).

Importantly, the study involved two levels of P-values. The first level of P-values for each SNP in the GWAS was calculated according to the genotypes of the participating individuals in relation to their CAD phenotype. For our purposes, these trait association P-values represent the statistical strengths of CAD associations, and produce the ranking of eSNPs according to their relevance to CAD. It has been previously observed that eSNPs are enriched for disease associations [36], [65]. Therefore, simply using eSNPs to determine pathway signals typically leads to false positives. In our study, we first removed SNPs that were not eSNPs, and used the remaining pool of eSNPs as the null background for subsequent enrichment tests. For instance, the background for adipose tissue comprised all the 59,979 non-redundant adipose eSNPs. This procedure was adopted for each tissue-specific eSNP set separately.

The gene set enrichment P-values represent the second level of P-values which reflect the degree of enrichment of high ranking disease-associated eSNPs within a given gene set as compared to the null distribution of randomly expected uniform distribution of all ranks. The enrichment of CAD association signals for each gene set (pathway or co-expression module) was estimated by the Kolmogorov-Smirnov (K-S) test and Fisher's exact test. The K-S test takes into account the total deviation of the observed ranks from the expectation and is therefore sensitive to a large number of weak GWAS signals. The Fisher's exact test detects if the top 5% of eSNPs based on their CAD association strength is over-represented among the eSNPs representing a gene set of interest (sensitive to a few strong signals). The final enrichment score was defined as the mean −log10 of K-S and Fisher P-values.

False discovery rates (FDR) were estimated by randomly permuting the CAD association P-values of the background eSNPs while keeping all other data structures intact. For a single permutation, FDR was estimated as the ratio between the number of permuted gene sets that exceeded a given enrichment score, and the observed number of gene sets that actually exceeded the score threshold. The final FDR was averaged over 1000 permutations for each tissue-specific eSNP set separately. Gene sets that satisfied FDR<20% in both Stage 1 and Stage 2 GWAS sets, and FDR<5% in the combined Stage 1+2 were considered statistically significant. As Stage 1 and Stage 2 were independent, the request for simultaneous satisfaction for these FDR cutoffs ensured the overall FDR to be <5%. SSEA was performed in R.

### Gene set overlaps and construction of supersets

A substantial number of gene sets overlapped based on their shared member genes, given that similar functional units of genes could be captured by the pathway databases and gene expression studies used in the study. To reduce the redundancy of the dataset, we collapsed overlapping CAD-associated gene sets into non-overlapping “supersets” (Figure 1B). When multiple gene sets are merged, the size of the resulting superset can grow very large. For this reason, we included only the core genes (that were shared with most of the constituent gene sets) in the final supersets.

For two gene sets A and B with different numbers of member genes, two overlap ratios were calculated: the proportion of genes in A that were also in B (*r_AB_*), and the proportion of genes in B that were also in A (*r_BA_*). We chose the formula *r* = (r_AB_×*r_BA_*)^0.5^ to describe the degree of overlap. Importantly, *r* is small whenever the sizes of A and B are substantially different, which discourages the merging of nested gene sets. We also required that Fisher's exact test for the number of shared genes was statistically significant (P<0.05 after Bonferroni correction).

We employed hierarchical clustering to define blocks of overlapping gene sets. First, the overlap matrix of the CAD-associated gene sets was estimated and all non-significant elements were set to zero. The overlaps were then converted to distances *d* = (1 - *r*). Clusters of overlapping gene sets were identified by the hclust() function in the R programming environment with a static cutoff at zero overlap. In the last step, the gene sets within clusters were merged and trimmed into supersets. The above procedure was repeated two times to reduce the maximum observed overlap below 20% between any two resulting supersets.

A size limit of 200 genes was chosen to trim the raw supersets down to the core genes that were shared across overlapping gene sets. This choice of optimal size was motivated by earlier SSEA analyses [37]. The least shared genes were successively removed until the next removal would have reduced the superset size below the 200-gene limit. Overlap ratios were re-calculated between the trimmed supersets before the next round of hierarchical clustering.

The functional categorization of each superset was based on the known pathways from the Gene Ontology and KEGG databases. We evaluated the over-representation of a functional category within the member genes of a superset with the Fisher's exact test. Significant functional categories (P<0.05 after Bonferroni correction) were used to annotate the functionality of each superset. If no significant annotation could be found, we labeled the superset as ‘Unknown’. A second round of SSEA was performed for the merged supersets to confirm that they captured the features of the pre-merged gene sets. Significance was determined at SSEA P<0.05 after Bonferroni-correction for the number of all original gene sets (n = 3539, not the number of supersets after merging) to ensure stringency.

### Bayesian network models of causal gene-gene interactions

The above SSEA analysis is able to identify a gene set that is likely to contain disease-causing genetic variation. To pinpoint the most influential regulatory genes, we utilized Bayesian network models of gene-gene interactions that take into account both the genotypes that affect gene expression (causal direction known), and the statistical relationships between gene expression levels (causal direction uncertain), using the established method by Zhu et al. [29], [67]. Bayesian network models from human and mouse studies were constructed based on genetics and gene expression data generated from multiple tissues from multiple previously published studies, each involving hundreds of individuals (details and references in Table S9). Human networks were obtained from adipose tissue, blood, and liver. Mouse networks were obtained from adipose tissue, liver, muscle, brain, and kidney.

Bayesian networks are directed acyclic graphs in which the edges of the graph are defined by conditional probabilities that characterize the distribution of states of each gene given the state of its parents [68]. The network topology defines a partitioned joint probability distribution over all genes in a network. The likelihood of a Bayesian network model given observed genomic data is determined using Bayes formula. For each dataset, 1000 Bayesian networks, each using different random seeds, were reconstructed using Monte Carlo Markov Chain simulation [69]. Bayesian Information Criteria was used to determine the model with the best fit for each network. From the resulting set of 1000 networks, edges that appeared in greater than 30% of the networks were used to define a consensus network for a given dataset. To infer causal directions between genes in a network, genetic information was used as priors by allowing genes with *cis*-eSNPs to be parent nodes of genes without *cis*-eSNPs and preventing genes without *cis*-eSNPs to be parents of genes with *ci*s-eSNPs [70]. Bayesian network provides a natural framework for integrating diverse data and reconstruct biological causal networks.

### Key driver analysis (KDA)

We used the key driver analysis (KDA) to determine the key regulatory genes of the CAD-associated gene supersets [12], [28], [29]. We defined a key driver as a gene that is connected to a large number of genes from a CAD-associated superset, compared to the expected number for a randomly selected gene within a Bayesian causal network. The basic idea of KDA is depicted in Figure 1C. First, one needs a network topology that defines links between pairs of genes. In this study, we used tissue-specific Bayesian networks that were constructed from large-scale genetic and genomic datasets from multiple previously published studies as described above. Second, a disease-related gene set is needed. In our study, this comes from the CAD-associated gene supersets (call it Gene Set S). We then tag each of the member genes in the Gene Set S within the network, as shown by the colored nodes in Figure 1C. For a gene in the network (call it Gene A), we then ask the following question: How many of Gene A's neighbors are members in the Gene Set S? If the proportion of member genes is higher than what could be expected for a random gene set, we define Gene A as a key driver for Gene Set S. The statistical significance of a key driver for a given CAD superset in a particular Bayesian network is determined by Fisher's exact test which assesses the enrichment of CAD genes in the candidate key driver's network neighborhood. Bonferroni-corrected p<0.05 was used to determine key drivers. As multiple networks were available for a number of tissues, we used two criteria to prioritize the five most important key driver genes. Firstly, we counted how many times a gene was a key driver in multiple networks (denoted as *N*). The consistency across networks was expressed as (*N* - 0.99) to strongly favor key genes that could be identified in at least two networks. The second criterion was based on the statistical significance of the key driver. In particular, the significance value was calculated as mean(-log ***P***), where ***P*** denotes the KDA significance P-values from each of the networks. The final ranking of genes was based on the product of the consistency and significance criteria. KDA was performed using R.

### Small interfering RNA (siRNA) experiments to test the regulatory role of candidate key driver genes

To test whether perturbing key drivers identified in our study indeed result in perturbations of CAD gene networks, we used siRNAs to knockdown the expression of novel key drivers in HAECs. HAECs were grown to 80% confluency on 0.1% gelatin coated culture plates in MCDB-131 complete medium (VEC technologies). Cells were transfected with siRNAs against each candidate key driver gene under investigation and a negative control (Cat. No. 1027280) at a final concentration of 40 nM with Lipofectamine 2000 reagent for 4 hours in Opti-MEM medium (Invitrogen). Three HAEC lines from different donors were used as biological replicates for each siRNA. Media was replaced with MCDB-131 and cells were lysed for RNA isolation after 24 hours. Whole transcriptome expression was assessed with Illumina HumanHT-12 v4 Expression BeadChip. We identified the differential expression of genes between cells transfected with the siRNAs targeting the candidate genes and the control siRNA using the limma package in R (2.14.0). Overlap between the differentially expressed genes in siRNA experiments and a CAD network of interest was assessed using Fisher's exact test.

## Supporting Information

Figure S1 QQ plots for the three new GWAS meta-analyses: Stage 1 (cyan), Stage 2 (green), and combined Stage 1+2 meta-analysis (orange).
(PNG)

Figure S2 Overlaps among CAD-associated gene sets. Hierarchical clustering of the CAD-associated gene sets A) before and B) after two rounds of merging and trimming based on overlapping ratios between gene sets. Red color indicates high overlaps and white color shows no overlap. Before merging, there are substantial overlaps among gene sets. After merging, the merged supersets are largely independent.
(PNG)

Figure S3 Effect of LD pruning on the number of distinct eSNPs. The numbers of eSNPs after LD pruning (Y axis) are plotted against the r^2^ LD values (X axis). As the LD cutoff becomes more stringent, the eSNP numbers gradually decrease, with the largest reduction of eSNPs occurring at r^2^ of 0.9.
(PNG)

Table S1 Significant GWAS loci from the combined Stage 1+2 meta-analysis and their relationship to known GWAS loci.
(XLSX)

Table S2 SSEA results for all significant canonical pathways. SSEA scores and FDR from Stage1+2 GWAS are shown.
(XLSX)

Table S3 Comparison of scores before and after incorporating three new large-scale blood eQTLs published between September 2013 and March 2014. The enrichment score was defined as the mean of negative log-transformed Kolmogorov-Smirnov and Fisher P-values for over-representation of high-ranking GWAS SNPs among the eSNPs that affect the expression of the pathway member genes. *FDR<20% in Stage 1 and 2 respectively, and FDR<5% in combined Stage 1+2.
(DOCX)

Table S4 SSEA results for all significant co-expression modules. SSEA scores and FDR from Stage1+2 GWAS are shown.
(XLSX)

Table S5 CAD enrichment scores for non-overlapping supersets after the merging of CAD-associated canonical pathways and co-expression modules. Annotations were summarized according to statistically significant over-representation of known pathways and processes. Supersets with at least one significant score in any tissue are included. *P<0.05 in either Fisher's exact test or Kolmogorov-Smirnov test after Bonferroni correction for the 3,539 original gene sets.
(DOCX)

Table S6 Top five GWAS signal genes and key regulator genes for selected CAD-associated supersets. A GWAS signal gene was defined as a gene that was functionally associated via one or more eQTL to the most statistically significant SNPs in the meta-analyzed GWAS. Key drivers were ascertained by combining key driver analyses of all available Bayesian networks, and taking into account both the consistency across datasets and the KDA statistics.
(DOCX)

Table S7 Top 5 key regulatory genes for CAD enriched supersets in tissue-specific gene regulatory networks based on key driver analysis. The genes within a tissue-specific table cell are ordered according to significance and consistency across multiple datasets when available. H = human, M = mouse.
(DOCX)

Table S8 Genome-wide association studies of CAD.
(DOCX)

Table S9 Data resources and references for eQTLs, co-expression networks, and Bayesian networks.
(DOCX)

Text S1 Algorithm to remove eSNPs of high LD from genetics of gene expression datasets.
(DOCX)
