# Supplementary figures and images for: Integrative Genomics Reveals Novel Molecular Pathways and Gene Networks for Coronary Artery Disease
Source: PLoS Genet. 2014 Jul 17;10(7):e1004502. doi: 10.1371/journal.pgen.1004502 (PMC4102418; doi:10.1371/journal.pgen.1004502)

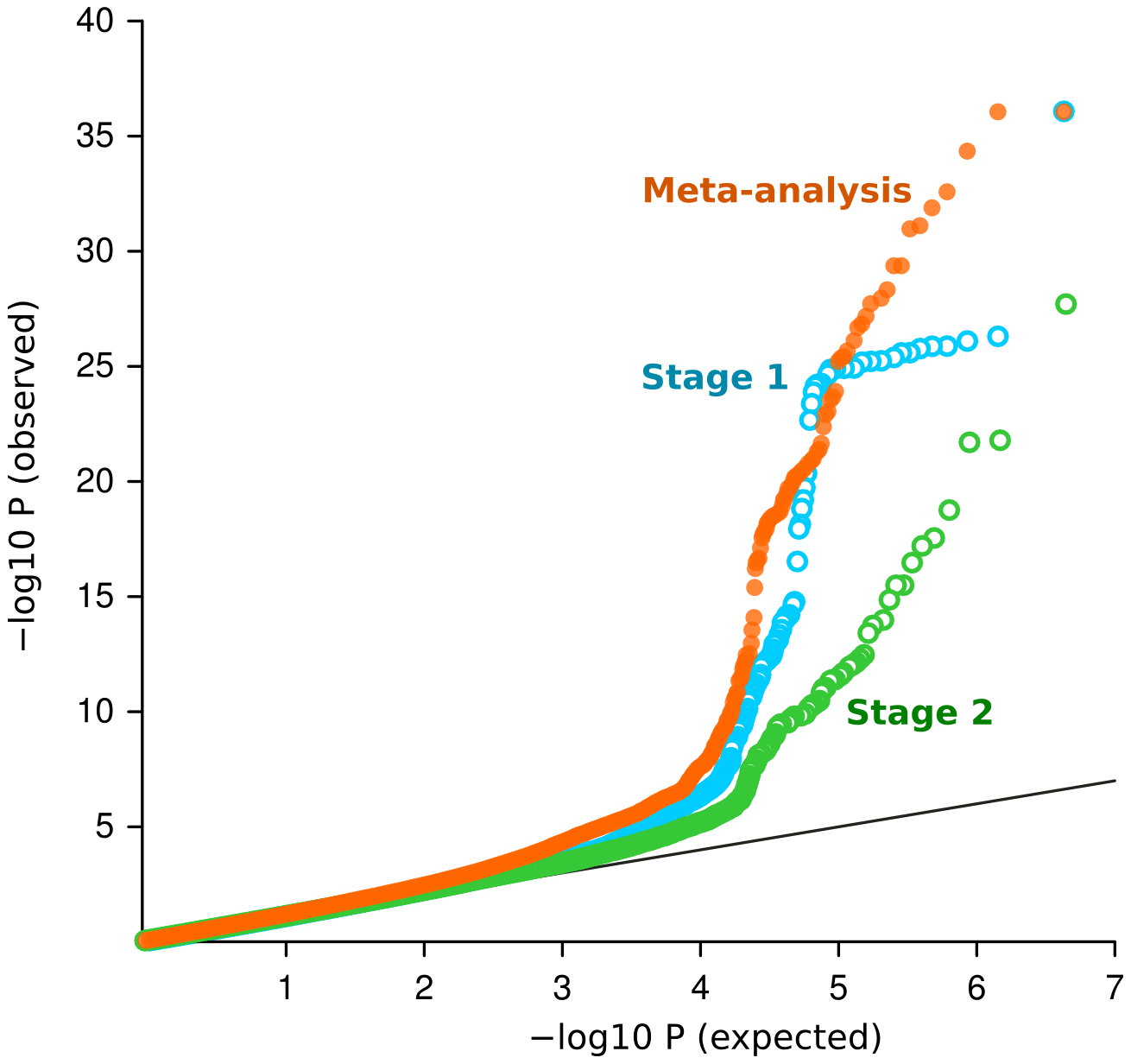

Supplement: Figure S1 — QQ plots for the three new GWAS meta-analyses: Stage 1 (cyan), Stage 2 (green), and combined Stage 1+2 meta-analysis (orange). (PNG) [file pgen.1004502.s001.png]

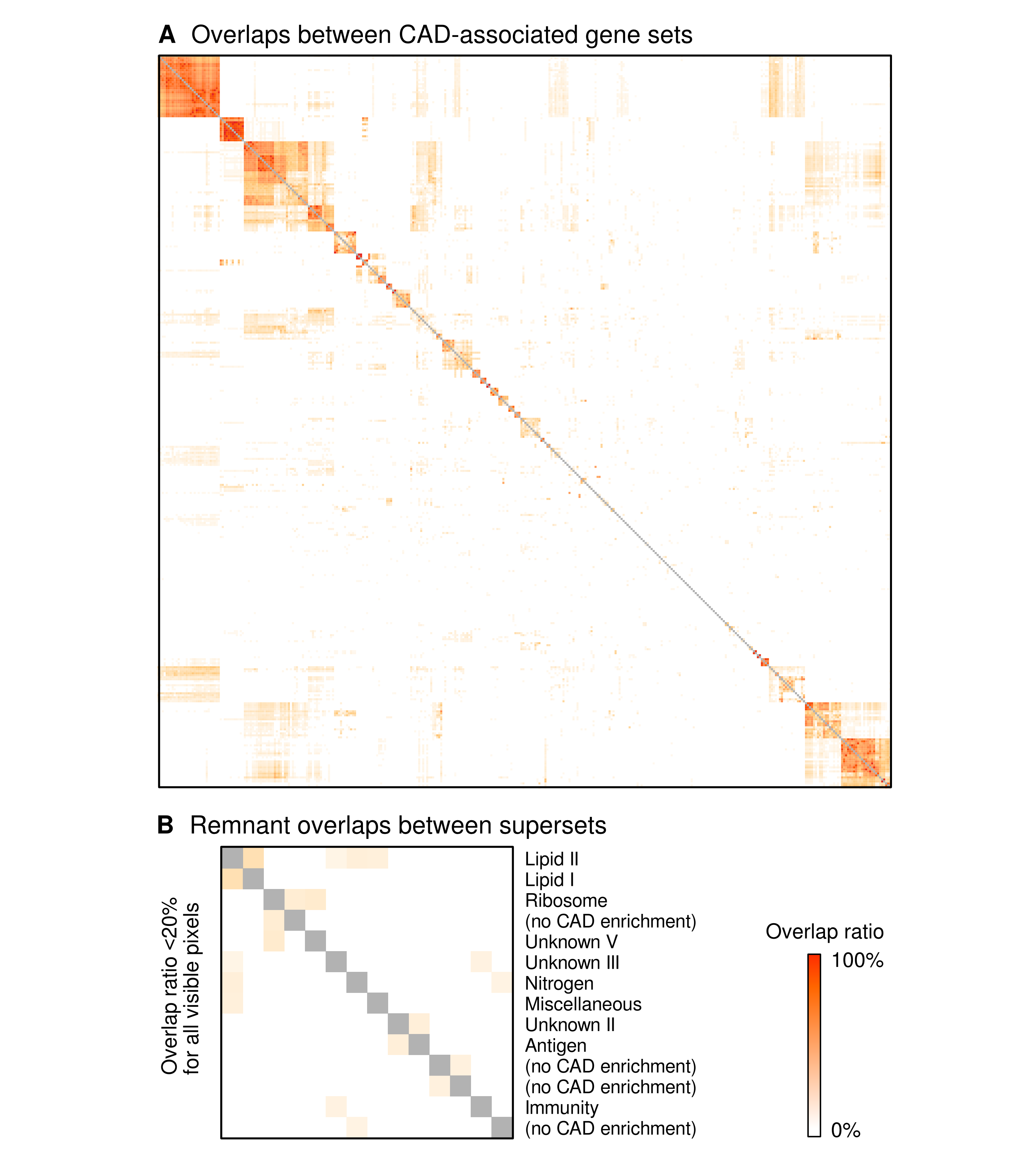

Supplement: Figure S2 — Overlaps among CAD-associated gene sets. Hierarchical clustering of the CAD-associated gene sets A) before and B) after two rounds of merging and trimming based on overlapping ratios between gene sets. Red color indicates high overlaps and white color shows no overlap. Before merging, there are substantial overlaps among gene sets. After merging, the merged supersets are largely independent. (PNG) [file pgen.1004502.s002.png]

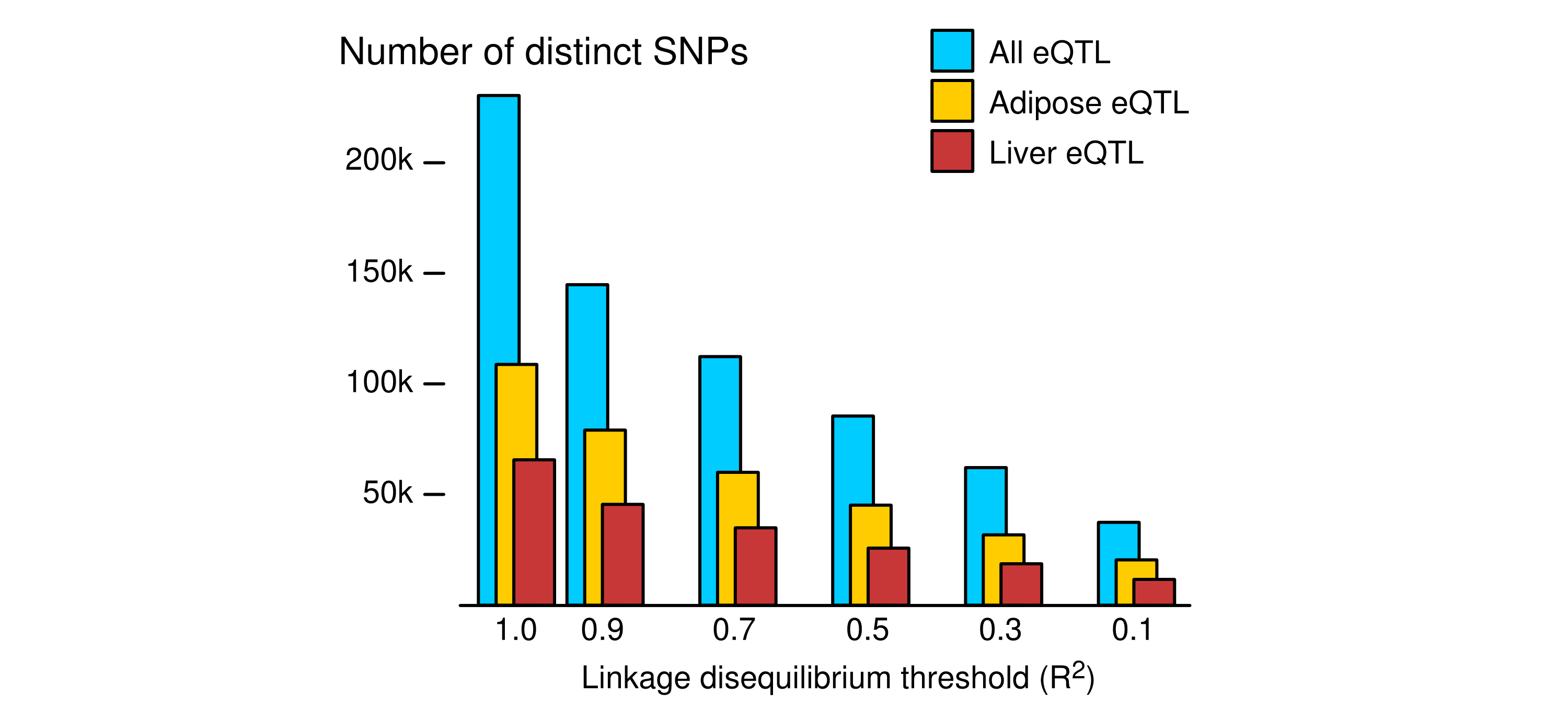

Supplement: Figure S3 — Effect of LD pruning on the number of distinct eSNPs. The numbers of eSNPs after LD pruning (Y axis) are plotted against the r2 LD values (X axis). As the LD cutoff becomes more stringent, the eSNP numbers gradually decrease, with the largest reduction of eSNPs occurring at r2 of 0.9. (PNG) [file pgen.1004502.s003.png]
