# Supplementary material for: Integrative Genomics Reveals Novel Molecular Pathways and Gene Networks for Coronary Artery Disease
Source: PLoS Genet. 2014 Jul 17;10(7):e1004502. doi: 10.1371/journal.pgen.1004502 (PMC4102418; doi:10.1371/journal.pgen.1004502)
Supplement: Table S3 — Comparison of scores before and after incorporating three new large-scale blood eQTLs published between September 2013 and March 2014. The enrichment score was defined as the mean of negative log-transformed Kolmogorov-Smirnov and Fisher P-values for over-representation of high-ranking GWAS SNPs among the eSNPs that affect the expression of the pathway member genes. *FDR<20% in Stage 1 and 2 respectively, and FDR<5% in combined Stage 1+2. (DOCX) [file pgen.1004502.s006.docx]

**Table S3. Comparison of scores before and after incorporating three new large-scale blood eQTLs published between September 2013 and March 2014.**

The enrichment score was defined as the mean of negative log-transformed Kolmogorov-Smirnov and Fisher P-values for over-representation of high-ranking GWAS SNPs among the eSNPs that affect the expression of the pathway member genes. *FDR < 20% in Stage 1 and 2 respectively, and FDR < 5% in combined Stage 1 & 2.

| **Pathways** | **OLD all eSNPs** | **NEW all**  **eSNPs** | **OLD blood** | **NEW blood** | **Significant in other tissues** |
| --- | --- | --- | --- | --- | --- |
| GWAS Catalog | 29.0* | 54.1* | 17.2* | 26.4* | all |
| CADGene | 12.0* | 15.7* | 1.3 | 6.8* | Adipose, liver |
| Metabolism of lipids and lipoproteins (Reactome) | 10.0* | 8.3* | 0.7 | 2.2* | adipose |
| Fatty acid metabolism (KEGG) | 5.2* | 4.2* | 0.4 | 1.5 | adipose |
| Recycling of bile acids and salts (Reactome) | 5.3* | 2.4 | - | 0.0 | liver |
| Immunoregulation between lymphoid and other cells (Reactome) | 9.4* | 10.5* | 1.8 | 4.7 | Adipose, liver |
| Antigen processing and presentation (KEGG) | 8.9* | 10.0* | 2.9* | 4.3* | Adipose, liver |
| Th1/Th2 differentiation (Biocarta) | 6.6* | 6.6* | 2.0 | 2.4 | Adipose, liver |
| Adhesion and diapesis of lymphocytes (Biocarta) | 3.3 | 3.1 | 0.8 | 1.2 | Adipose, HAEC |
| Adhesion and diapedesis of granulocytes (Biocarta) | 3.3 | 2.2 | 0.9 | 0.7 | Adipose, HAEC |
| VEGF, hypoxia and angiogenesis (Biocarta) | 4.5* | 2.9 | 3.2 | 1.8 | Adipose, liver |
| Erythropoietin mediated neuroprotection through NF-kB (Biocarta) | 3.0* | 4.6* | 2.2 | 3.4* | Adipose, liver |
| Hypoxia-inducible factor in the cardiovascular system (Biocarta) | 1.5 | 0.3 | 1.9 | 0.2 | Adipose |
| Notch-HLH transcription (Reactome) | 2.5 | 1.5 | - | 0.7 | Adipose |
| NRAGE signals death through JNK (Reactome) | 3.2* | 6.0* | 5.3* | 4.9* | Adipose |
| EGF signaling pathway (Biocarta) | 1.7 | 2.1 | 1.8 | 2.0 | Adipose |
| G1/S transition (Reactome) | 1.2 | 1.0 | 0.1 | 0.3 | Liver |
| Double-strand break repair (Reactome) | 3.1* | 2.9* | 3.3* | 1.5 |  |
| Spliceosome (KEGG) | 1.7 | 5.5* | 5.9* | 4.5* |  |
| Metabolism of proteins (Reactome) | 2.1* | 4.9* | 2.6 | 3.5 |  |
| Proteasome (KEGG) | 0.9 | 0.7 | 0.2 | 0.2 | Liver |
| Post-translational protein modifications (Reactome) | 1.3 | 2.4 | 0.7 | 1.3 | Adipose |
| Bioactive peptide induced signaling (Biocarta) | 3.2* | 4.8* | 1.5 | 2.4 | Adipose |
| PPAR signaling pathway (KEGG) | 3.1* | 2.4* | 0.6 | 1.7 | Adipose |
| Glycine, serine and threonine metabolism (KEGG) | 2.4* | 3.9* | 2.4 | 3.6 | Adipose |
