# Supplementary material for: Integrative Genomics Reveals Novel Molecular Pathways and Gene Networks for Coronary Artery Disease
Source: PLoS Genet. 2014 Jul 17;10(7):e1004502. doi: 10.1371/journal.pgen.1004502 (PMC4102418; doi:10.1371/journal.pgen.1004502)
Supplement: Table S5 — CAD enrichment scores for non-overlapping supersets after the merging of CAD-associated canonical pathways and co-expression modules. Annotations were summarized according to statistically significant over-representation of known pathways and processes. Supersets with at least one significant score in any tissue are included. *P<0.05 in either Fisher's exact test or Kolmogorov-Smirnov test after Bonferroni correction for the 3,539 original gene sets. (DOCX) [file pgen.1004502.s008.docx]

| **Table S5. CAD enrichment scores for non-overlapping supersets after the merging of CAD-associated canonical pathways and co-expression modules.** Annotations were summarized according to statistically significant over-representation of known pathways and processes. Supersets with at least one significant score in any tissue are included. *P < 0.05 in either Fisher's exact test or Kolmogorov-Smirnov test after Bonferroni correction for the 3,539 original gene sets. | | | | | | | |
| --- | --- | --- | --- | --- | --- | --- | --- |
| **Superset** | **Number of genes** | **Overlap with known processes** | **All eSNPs** | **Adipose** | **Liver** | **Blood** | **HAEC** |
| Lipid I | 217 | Lipid, fatty acid and steroid metabolism; oxidoreductase; PPAR signaling; mitochondrial beta-oxidation; branched-chain amino acid degradation; cholesterol biosynthesis; unsaturated fatty acid biosynthesis | 5.4* | 9.4* | 0.4 | 0.4 | 0.7 |
| Lipid II | 234 | Lipid, fatty acid and steroid metabolism; oxidoreductase; vesicles; xenobiotics; complement and coagulation system | 10.3* | 11.0* | 1.9 | 1.8 | 0.1 |
| Antigen | 221 | Human leukocyte antigens; bone resorption | 10.3* | 9.5* | 8.6* | 3.7 | 1.1 |
| Immunity | 110 | Wound and inflammatory responses; cell activation | 6.1* | 7.4* | 8.7* | 1.5 | 1.4 |
| Signaling I | 188 | MAPK and other protein kinases; phosphorylation; cell differentiation, cycle and motility; B-cell receptor; various other signaling cascades | 9.1* | 14.3* | 4.2 | 2.4 | 2.0 |
| Signaling II | 293 | GTPase; actin cytoskeleton and cell motility; (lytic) vacuole | 4.2 | 5.6* | 0.9 | 3.8 | 1.0 |
| Proteolysis | 115 | Proteolysis; ubiquitination; cell cycle; proteasome; TGF-beta signaling | 1.8 | 1.6 | 5.9* | 0.1 | 0.2 |
| Mitochondrion | 150 | Oxidative phosphorylation | 6.3* | 6.6* | 2.3 | 1.2 | 0.2 |
| Nitrogen | 198 | Amine and amino acid metabolism; urea cycle | 7.0* | 3.4 | 6.7* | 2.1 | 4.8* |
| Ribosome | 188 | Ribosome; protein biosynthesis | 7.1* | 6.0* | 0.6 | 2.8 | 0.9 |
| Transcription | 472 | GATA-X transcription factor | 7.4* | 3.3 | 3.1 | 0.4 | 1.6 |
| RNA | 123 | RNA binding, processing and splicing; spliceosome | 2.0 | 0.9 | 0.3 | 6.2* | 0.6 |
| Muscle | 515 | Muscle contraction; synaptic transmission | 4.9* | 8.5* | 2.1 | 0.2 | 0.2 |
| Miscellaneous | 126 | Lipid, fatty acid and steroid metabolism; amine and amino acid metabolism; RNA metabolism; prostaglandins; eicosanoid signaling | 2.8 | 5.4* | 0.6 | 0.2 | 0.6 |
| Unknown I | 127 | - | 3.5 | 7.4* | 4.4 | 1.2 | 0.2 |
| Unknown II | 140 | - | 2.9 | 6.4* | 3.9 | 2.2 | 0.2 |
| Unknown III | 56 | - | 4.0 | 6.8* | 0.1 | 1.0 | 0.6 |
| Unknown IV | 164 | - | 4.4 | 9.4* | 6.7* | 2.2 | 1.7 |
| Unknown V | 529 | - | 9.6* | 7.6* | 3.6* | 1.2 | 0.5 |
| Unknown VI | 80 | - | 3.0 | 7.0* | 2.3 | 1.1 | 0.4 |
| Unknown VII | 117 | - | 3.4 | 5.5* | 0.8 | 0.2 | 2.1 |
| Unknown VIII | 71 | - | 2.2 | 0.9 | 4.9* | 1.5 | - |
