# Supplementary material for: Integrative Genomics Reveals Novel Molecular Pathways and Gene Networks for Coronary Artery Disease
Source: PLoS Genet. 2014 Jul 17;10(7):e1004502. doi: 10.1371/journal.pgen.1004502 (PMC4102418; doi:10.1371/journal.pgen.1004502)
Supplement: Table S6 — Top five GWAS signal genes and key regulator genes for selected CAD-associated supersets. A GWAS signal gene was defined as a gene that was functionally associated via one or more eQTL to the most statistically significant SNPs in the meta-analyzed GWAS. Key drivers were ascertained by combining key driver analyses of all available Bayesian networks, and taking into account both the consistency across datasets and the KDA statistics. (DOCX) [file pgen.1004502.s009.docx]

| **Table S6. Top five GWAS signal genes and key regulator genes for selected CAD-associated supersets.** A signal gene was defined as a gene that was functionally associated via one or more eQTL to the most statistically significant SNPs in the meta-analyzed GWAS. Key drivers were ascertained by combining key driver analyses of all available Bayesian networks, and taking into account both the consistency across datasets and the KDA statistics. | | |
| --- | --- | --- |
| **Superset** | **GWAS signal genes** | **Key regulator genes** |
| Lipid I | SREBF1, LPL, LDLR, CYP4A11, ME1 | DCI, SQLE, ETHDH, SLC22A5, EHHADH |
| Lipid II | TMEM116, TMEM27, MAT1A, LRRC19, NAT2 | GC, CES3, PZP, HGR, PLG |
| Immunity | CTSS, HLA-B, OAS1, HLA-DRB1, HLA-DQB1 | PTPRC, NCKAP1L, FCGR1A, FYB, FCER1G |
| Antigen | CD2AP, AS3MT, HCG4, TAF11, FLOT1 | VPS52, PPIL1, GLO1, GFER, DECR2 |
| Signaling I | MAPKAPK5, EDNRA, CDKN1A, CAMK2G, ARNT | CHL1, USP9X, PAK2, PDLIM5 |
| Signaling II | TCTN1, SIDT2, KIAA0913, CCDC92, SPG7 | DENND4B, PDPN, COL4A2, COL6A2 |
| Proteolysis | PSMA5, PSMA4, CDKN1A, CDC27, POU5F1 | FIGNL1, CDCA8, BIRC5, NCAPG, RAD51 |
| Mitochondrion | PSMA5, CEP70, USMG5, AXL, ATP6V1G2 | TOMM7, C14orf156, APH1B, PIN4, MAP3K9 |
| Nitrogen | SLC22A3, ALDH2, SFXN2, SRR, SHMT1 | CPS1, GNMT, ASS1, ADFP, GOT1 |
| Ribosome | RPL6, BSDC1, LASS2, MAP3K4, TRPC4AP | NFIX, RPS27, RHBDD2, NUP210, RPL12 |
| Transcription | TMEM27, SFTPC, MUC20, PECAM1, DHX36 | - |
| RNA | LSM4, SNRPC, USP39, DHX38, HSPA1A | - |
| Muscle | TAGLN, TMEM116, SFXN2, MYH11, C16orf45 | MYH11, MYL9, CNN1, MYOCD, PCP4 |
| Miscellaneous | MAT1A, CYP4A11, VARS2, GGT7, CARS2 | - |
| Unknown I | NT5C2, SURF6, ARL3, LMO4, TIE1 | DNAJC7, UBE2S, ALG8, ZC3H7B, PRMT1 |
| Unknown II | ALS2CR13, TMEM116, C10orf26, CEACAM3, NM_152451 | CEBPD, SGK1, SLC10A6, KCNA5, MAP3K6 |
| Unknown III | CYP4A11, SULT1E1, TRIB3, IL5RA, OR4M1 | CXCR6, C12orf50, TBC1D10C, PSCDBP, STAT4 |
| Unknown IV | TMEM116, MLH3, EIF2AK2, C1orf64, HOXA2 | TMEM141, GFRAL, CHRM5, TLX1 |
| Unknown V | SORT1, TOM1L2, BSDC1, VPS29, DNMT1 | KIAA0652, FASN, USP20, DENND4B, RUSC2 |
| Unknown VI | TMEM116, SOX7, C3orf57, TEX14, SMARCE1 | - |
| Unknown VII | TAF13, PSORS1C1, STRN, LHX5, C6orf189 | - |
| Unknown VIII | PSRC1, ZBTB16, WDR76, SSTR2, TITF1 | LHX9, CLK3, RUNDC3A, NM144610 |
