# Supplementary material for: Integrative Genomics Reveals Novel Molecular Pathways and Gene Networks for Coronary Artery Disease
Source: PLoS Genet. 2014 Jul 17;10(7):e1004502. doi: 10.1371/journal.pgen.1004502 (PMC4102418; doi:10.1371/journal.pgen.1004502)
Supplement: Table S7 — Top 5 key regulatory genes for CAD enriched supersets in tissue-specific gene regulatory networks based on key driver analysis. The genes within a tissue-specific table cell are ordered according to significance and consistency across multiple datasets when available. H = human, M = mouse. (DOCX) [file pgen.1004502.s010.docx]

| **Table S7. Top 5 key regulatory genes for CAD enriched supersets in tissue-specific gene regulatory networks based on key driver analysis.** The genes within a tissue-specific table cell are ordered according to significance, and consistency across multiple datasets when available. H=human, M=mouse. | | | | | | |
| --- | --- | --- | --- | --- | --- | --- |
| **Superset** | **Adipose (H & M)** | **Brain (M)** | **Kidney (M)** | **Liver (H & M)** | **Muscle (M)** | **Within top 5 in multiple tissues** |
| Lipid I | ETFDH, CPT2, FDPS, ECHS1, IDI1 | KNG1, HGD, RGN, CES3, APOC4 | ACOT2, PDK4, EHHADH, HMGCS2, ANGPTL4 | ACOT2, DCI, SLC22A5, SQLE, ALDH3A2 | EPHX2, APOC1, KNG1, MAT1A, ACAA2 | ACOT2, KNG1 |
| Lipid II | PLG, GC, MBL2, SERPINC1, CES3 | KNG1, PZP, HRG, GC, CFI | PLG, F2, CYP3A5, ARG1, ALB | VTN, CFI, PLG, SERPINF2, F2 | AHSG, SERPINC1, GC, ASGR1, PLG | CFI, F2, GC, PLG, SERPINC1 |
| Immunity | FCER1G, HCK, AIF1, LY86, FERMT3 | FCER1G, FYB, PSMB9, ENST00000361050, BCL2A1 | FYB, C1orf38, FCER1G, CORO1A, NCKAP1L | CSF1R, ITGB2, NCKAP1L, C1orf38, HCK | HCK, SASH3, CTSS, ENST00000361050, PTPRC | C1orf38, ENST00000361050, FCER1G, FYB, HCK, NCKAP1L |
| Antigen | GLO1, VPS52, HLA-DRA, PPIL1, CDSN | PPIL1, CYP4F12, RSPH1, GLO1, VPS52 | HLA-DQA1, GLO1, HLA-E, SKIV2L, ZNF627 | VPS52, GLO1, GFER, GNPTG, ZNF627 | GFER, PPIL1, VPS52, DECR2, SLC37A1 | GFER, GLO1, PPIL1, VPS52, ZNF627 |
| Signaling I | - | CHL1, USP9X, PAK2, PDLIM5 | - | - | - | - |
| Signaling II | - | - | - | - | PDPN, COL4A2, COL6A2 | - |
| Proteolysis | FIGNL1, NCAPG, RAD51, CDCA8, CDCA5 | - | - | CDCA8, CENPM, FIGNL1, BIRC5, UHRF1 | PRC1, BIRC5, CENPE, SMC2, RRM2 | BIRC5, CDCA8, FIGNL1 |
| Mitochondrion | C14orf156, USMG5, TOMM7, TIMM8B, NDUFAB1 | APH1B, C14orf156, PIN4, UQCRB, C14orf2 | - | PIGT, LRIT3, MRPS16, PDZD2, TOMM7 | MAP3K9, MIER2, TOMM7, RTKN, C14orf156 | C14orf156, TOMM7 |
| Nitrogen | SLC22A1, F2, PROC, CPS1, AASS | KNG1, FETUB, C9, CYP3A5, AZGP1 | - | CPS1, ADFP, ASS1, GOT1, LMAN2L | SERPINC1, TTC36, ALDOB, F13B, HGD | CPS1 |
| Ribosome | RPS27, RPL12, RPL31, MIF4GD, NOTCH1 | - | - | - | NFIX, RHBDD2, NUP210, WBP2, PDE4A | - |
| Muscle | MYH11, CNN1, MYOCD, PCP4, PDLIM3 | CNTN4, SLC26A4, EPHA4, TAGLN, LHX2 | KCNMB4, DBC1, CHGB | DLK1, C2orf88, LIPN, RLN3, BEX1 | KIF5A, SNAP25, SLC6A1, SULT4A1, MLC1 | - |
| Unknown I | SLC10A6, FKBP5, KCNA5, C10orf10, MAP3K6 | SGK1, NM_014859, PLEKHF1, CEBPD, NFKBIA | KCNK5, GADD45G, PLK3, ZFP36, DUSP1 | CEBPD, RASGEF1B, DDIT4, MAFB, ERRFI1 | ARRDC2, MAP3K6, NM_014859, SLC10A6, DDIT4 | CEBPD, DDIT4, KCNA5, MAP3K6, NM_014859, SLC10A6 |
| Unknown II | RAB35, ZC3H7B, GPR182, PHF1, TKT | TKT, ZC3H7B, TRAPPC3, PSPC1, DNAJC7 | GIGYF2, ARPC3, RAB35, AK055378, SH3D19 | DNAJC7, SH3D19, ALG8, TRAPPC3, UBE2S | UBE2S, RAB5C, BC007901, DNAJC7, ALG8 | ALG8, DNAJC7, RAB35, SH3D19, TKT, TRAPPC3, UBE2S, ZC3H7B |
| Unknown III | CXCR6, TBC1D10C, PSCDBP, SH2D1A | - | - | C12orf50, STAT4 | - | - |
| Unknown IV | - | - | TMEM141, GFRAL, CHRM5, TLX1 | - | - | - |
| Unknown V | SLC2A4, UNC84B | FASN, USP20, BIN1, SAMD14, DENND4B | - | - | - |  |
| Unknown VIII | - | - | - | - | LHX9, RUNDC3A, CLK3, NM_144610 | - |
