# Supplementary material for: Integrative Genomics Reveals Novel Molecular Pathways and Gene Networks for Coronary Artery Disease
Source: PLoS Genet. 2014 Jul 17;10(7):e1004502. doi: 10.1371/journal.pgen.1004502 (PMC4102418; doi:10.1371/journal.pgen.1004502)
Supplement: Table S8 — Genome-wide association studies of CAD. (DOCX) [file pgen.1004502.s011.docx]

| **Table S8. Genome-wide association studies of CAD.** | | | | |
| --- | --- | --- | --- | --- |
| GWAS name | Number of cases/controls | Age (mean±SD) of  cases/controls | % male in  cases/controls | % myocardial infarction in cases |
| *Stage 1* | | | | |
| OHGS_A | 921/994 | 48.2±7.0/74.9±4.9 | 78.1/54.6 | 54.6 |
| OHGS_CCGB-B | 2,688/1,819 | 49.8±7.7/74.8±5.4 | 75.1/49.0 | 59.8 |
| DUKE_2 | 1,200/648 | 56.7±9.7/63.3±8.7 | 69.4/42.0 | 48.0 |
| GerMIFs I | 875/1,644 | 50.2±7.8/62.6±10.0 | 50.6/49.2 | 100 |
| GerMIFs II | 1,222/1,298 | 51.4±7.5/51.2±11.9 | 66.9/51.7 | 100 |
| GerMIFs III (KORA) | 1,157/1,748 | 58.6±8.7/55.9±10.7 | 79.9/51.1 | 100 |
| WTCCC | 1,926/2,938 | 49.8±7.7/N/A | 79.3/50.0 | 71.5 |
| *Total Stage 1* | 9,889/11,089 |  | | |
| *Stage 2* | | | | |
| ADVANCE | 278/312 | 45.8±6.2/45.3±5.7 | 42.1/41.0 | 50.4 |
| CADomics | 2,078/2,952 | 60.8±10.1/55.3±10.8 | 78.1/49.5 | 58.3 |
| CHARGE | 2,287/22,024 | 60.0±7.9/63.1±8.0 | 66.6/40.4 | 48.0 |
| deCODE CAD | 6,640/27,611 | 74.8±11.8/53.7±21.5 | 63.7/38.1 | 54.7 |
| LURIC/AtheroRemo 1 | 652/213 | 61.0±11.8/58.3±12.1 | 79.7/54.0 | 71.9 |
| LURIC/AtheroRemo 2 | 486/296 | 63.7±9.4/56.4±12.7 | 76.6/51.4 | 79.0 |
| MedStar | 874/447 | 48.9±6.4/59.7±8.9 | 67.0/45.4 | 48.1 |
| MIGen | 1,274/1,407 | 42.4±6.6/43.0±7.8 | 62.8/60.1 | 100 |
| PennCATH | 933/468 | 52.7±7.6/61.7±9.6 | 76.3/48.1 | 50.3 |
| *Total Stage 2* | 12,501/55,730 |  | | |
| *Total Stage 1 & 2* | 25,491/66,819 |  | | |
