# Supplementary material for: Integrative Genomics Reveals Novel Molecular Pathways and Gene Networks for Coronary Artery Disease
Source: PLoS Genet. 2014 Jul 17;10(7):e1004502. doi: 10.1371/journal.pgen.1004502 (PMC4102418; doi:10.1371/journal.pgen.1004502)
Supplement: Table S9 — Data resources and references for eQTLs, co-expression networks, and Bayesian networks. (DOCX) [file pgen.1004502.s012.docx]

| **Table S9. Data resources and references for eQTLs, co-expression networks, and Bayesian networks.** | | | | | |
| --- | --- | --- | --- | --- | --- |
| Tissue | Species | eSNP data | Co-expr. modules | Bayesian networks | Dataset |
| Aortic endothelial cells | Human | yes | yes | no | 147 heart transplant donors [1] |
| Adipose tissue | Human | yes | yes | yes | 1,675 individuals from two Icelandic cohorts [2] |
|  | Human | yes | yes | no | 1,008 obese patients [3] |
|  | Human | yes | no | no | 150 female twins [4] |
|  | Mouse | - | yes | yes | C57BL/6J x A/J mouse cross [5] |
|  | Mouse | - | yes | yes | C57BL/6J x C3H ApoE -/- mouse cross [6,7] |
|  | Mouse | - | yes | yes | C57BL/6J x C3H wildtype mouse cross [8] |
|  | Mouse | - | yes | yes | C57BL/6J x BTBR Lepob mouse cross [9] |
| Blood | Human | yes | yes | yes | 1,675 individuals from two Icelandic cohorts [2] |
|  |  | yes | no | no | 1,469 unrelated individuals [10] |
| Brain | Mouse | - | yes | yes | C57BL/6J x A/J mouse cross [5] |
|  | Mouse | - | yes | yes | C57BL/6J x C3H ApoE -/- mouse cross [6,7] |
|  | Mouse | - | yes | yes | C57BL/6J x BTBR Lepob mouse cross [9] |
| Fibroblasts | Human | yes | no | no | Umbilical cords of 85 Western European individuals [11] |
| Heart | Mouse | - | yes | no | C57BL/6J x A/J mouse cross [5] |
| Islet cells | Mouse | - | yes | no | C57BL/6J x BTBR Lepob mouse cross [9] |
| Kidney | Mouse | - | yes | yes | C57BL/6J x A/J mouse cross [5] |
| Liver | Human | yes | yes | yes | 427 individuals [8] |
|  | Human | yes | yes | no | 1,008 obese patients [3] |
|  | Mouse | - | yes | yes | C57BL/6J x A/J mouse cross [5] |
|  | Mouse | - | yes | yes | C57BL/6J x C3H ApoE -/- mouse cross [6,7] |
|  | Mouse | - | yes | yes | C57BL/6J x C3H wildtype mouse cross [8] |
|  | Mouse | - | yes | yes | C57BL/6J x BTBR Lepob mouse cross [9] |
| Lymphoblasts | Human | yes | no | no | Umbilical cords of 85 Western European individuals [11] |
|  | Human | yes | no | no | 400 children of families with a proband with asthma [12] |
|  | Human | yes | no | no | 60 HapMap participants of European descent [13] |
|  | Human | yes | no | no | 270 HapMap participants [14] |
|  | Human | yes | no | no | 726 HapMap3 participants [15] |
|  | Human | yes | no | no | 30 European and 30 Yoruba HapMap participants [16] |
|  | Human | yes | no | no | 150 female twins [4] |
| Monocytes | Human | yes | no | no | 1,490 unrelated individuals [17] |
| Muscle | Mouse | - | yes | yes | C57BL/6J x A/J mouse cross [5] |
|  | Mouse | - | yes | yes | C57BL/6J x C3H ApoE -/- mouse cross [6,7] |
|  | Mouse | - | yes | yes | C57BL/6J x C3H wildtype mouse cross [8] |
|  | Mouse | - | yes | yes | C57BL/6J x BTBR Lepob mouse cross [9] |

**References**

1. Erbilgin A, Civelek M, Romanoski CE, Pan C, Hagopian R, et al. (2013) Identification of CAD candidate genes in GWAS loci and their expression in vascular cells. J Lipid Res 54: 1894–1905. doi:10.1194/jlr.M037085.

2. Emilsson V, Thorleifsson G, Zhang B, Leonardson AS, Zink F, et al. (2008) Genetics of gene expression and its effect on disease. Nature 452: 423–428. doi:10.1038/nature06758.

3. Greenawalt DM, Dobrin R, Chudin E, Hatoum IJ, Suver C, et al. (2011) A survey of the genetics of stomach, liver, and adipose gene expression from a morbidly obese cohort. Genome Res 21: 1008–1016. doi:10.1101/gr.112821.110.

4. Nica AC, Parts L, Glass D, Nisbet J, Barrett A (2011) The architecture of gene regulatory variation across multiple human tissues: the MuTHER study. PLoS Genet.

5. Derry JMJ, Zhong H, Molony C, MacNeil D, GuhaThakurta D, et al. (2010) Identification of genes and networks driving cardiovascular and metabolic phenotypes in a mouse F2 intercross. PLoS ONE 5: e14319. doi:10.1371/journal.pone.0014319.

6. Wang SS, Schadt EE, Wang H, Wang X, Ingram-Drake L, et al. (2007) Identification of pathways for atherosclerosis in mice: integration of quantitative trait locus analysis and global gene expression data. Circ Res 101: e11–e30. doi:10.1161/CIRCRESAHA.107.152975.

7. Yang X, Schadt EE, Wang S, Wang H, Arnold AP, et al. (2006) Tissue-specific expression and regulation of sexually dimorphic genes in mice. Genome Res 16: 995–1004. doi:10.1101/gr.5217506.

8. Schadt EE, Molony C, Chudin E, Hao K, Yang X, et al. (2008) Mapping the genetic architecture of gene expression in human liver. PLo
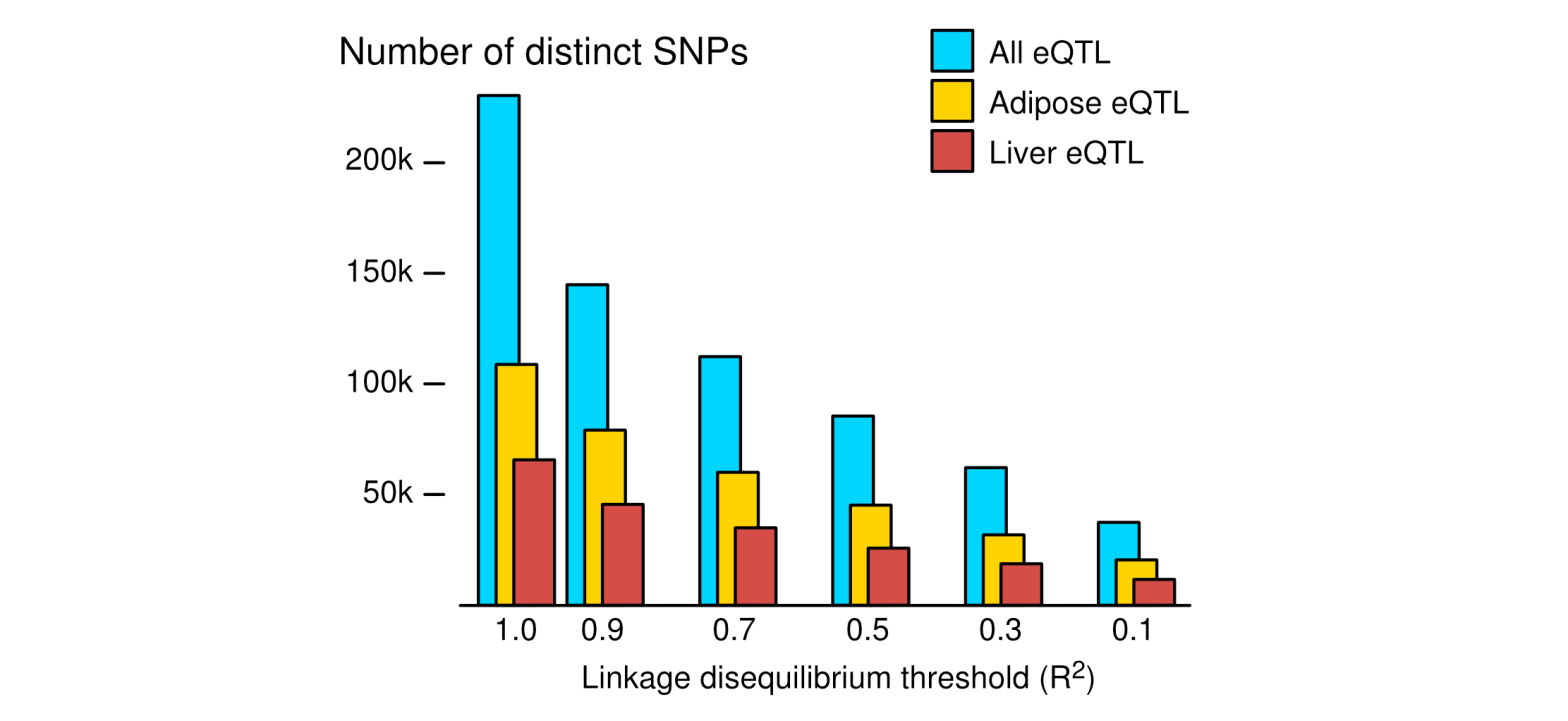

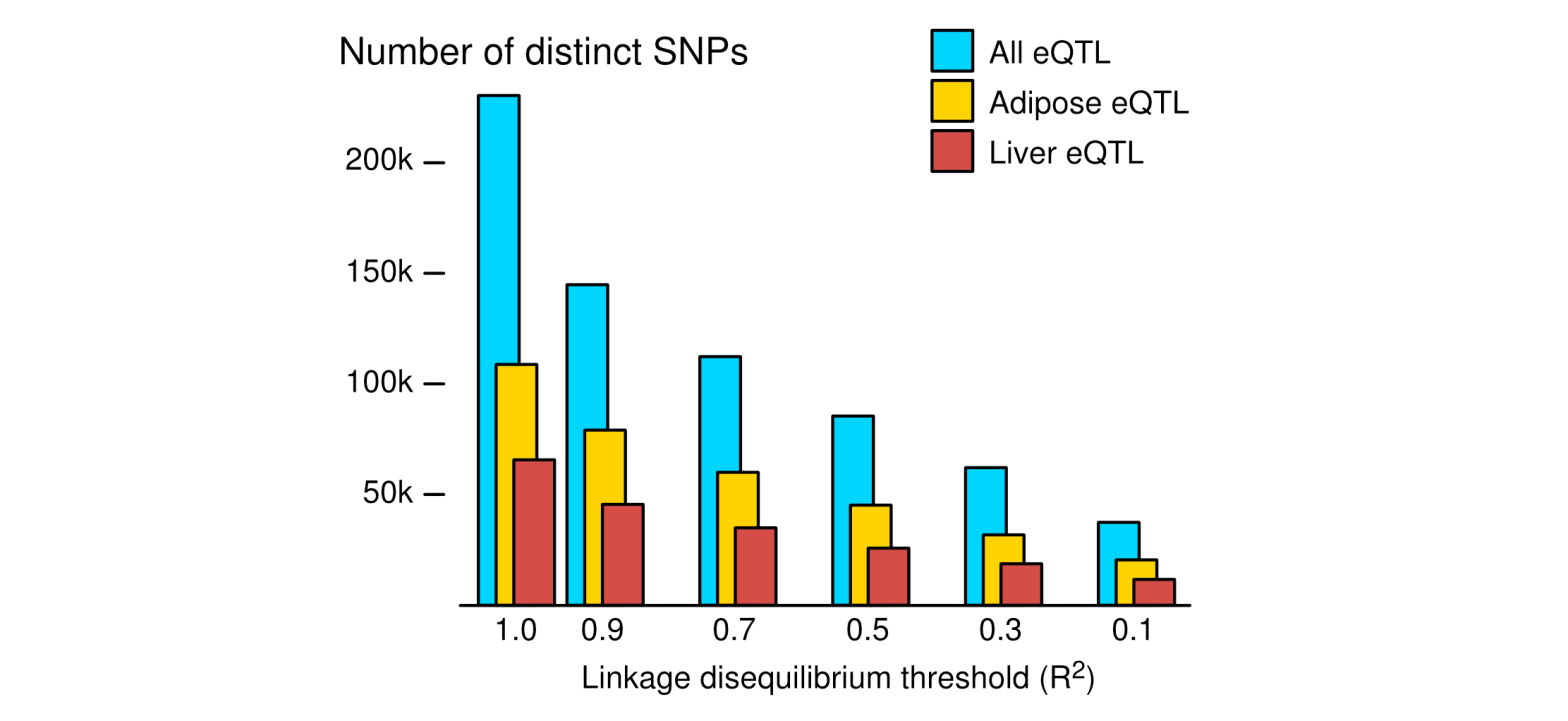
S Biol 6: e107. doi:10.1371/journal.pbio.0060107.

9. Tu ZZ, Keller MPM, Zhang CC, Rabaglia MEM, Greenawalt DMD, et al. (2012) Integrative analysis of a cross-loci regulation network identifies App as a gene regulating insulin secretion from pancreatic islets. PLoS Genet 8: e1003107–e1003107. doi:10.1371/journal.pgen.1003107.

10. Fehrmann R, Jansen RC, Veldink JH, Westra HJ (2011) Trans-eQTLs reveal that independent genetic variants associated with a complex phenotype converge on intermediate genes, with a major role for the HLA. PLoS Genet.

11. Dimas AS, Deutsch S, Stranger BE, Montgomery SB, Borel C, et al. (2009) Common regulatory variation impacts gene expression in a cell type-dependent manner. Science 325: 1246–1250. doi:10.1126/science.1174148.

12. Dixon AL, Liang L, Moffatt MF, Chen W, Heath S, et al. (2007) A genome-wide association study of global gene expression. Nat Genet 39: 1202–1207. doi:10.1038/ng2109.

13. Montgomery SB, Sammeth M, Gutierrez Arcelus M, Lach RP, Ingle C, et al. (2010) Transcriptome genetics using second generation sequencing in a Caucasian population. Nature 464: 773–777. doi:10.1038/nature08903.

14. Stranger BE, Nica AC, Forrest MS, Dimas A, Bird CP, et al. (2007) Population genomics of human gene expression. Nat Genet 39: 1217–1224. doi:10.1038/ng2142.

15. Stranger BEB, Montgomery SBS, Dimas ASA, Parts LL, Stegle OO, et al. (2012) Patterns of cis regulatory variation in diverse human populations. PLoS Genet 8: e1002639–e1002639. doi:10.1371/journal.pgen.1002639.

16. Duan SS, Huang RSR, Zhang WW, Bleibel WKW, Roe CAC, et al. (2008) Genetic Architecture of Transcript-Level Variation in Humans. Am J Hum Genet 82: 13–13. doi:10.1016/j.ajhg.2008.03.006.

17. Zeller TT, Wild PP, Szymczak SS, Rotival MM, Schillert AA, et al. (2010) Genetics and beyond--the transcriptome of human monocytes and disease susceptibility. PLoS ONE 5: e10693–e10693. doi:10.1371/journal.pone.0010693.
