# Supplementary material for: Integrative Genomics Reveals Novel Molecular Pathways and Gene Networks for Coronary Artery Disease
Source: PLoS Genet. 2014 Jul 17;10(7):e1004502. doi: 10.1371/journal.pgen.1004502 (PMC4102418; doi:10.1371/journal.pgen.1004502)
Supplement: Text S1 — Algorithm to remove eSNPs of high LD from genetics of gene expression datasets. (DOCX) [file pgen.1004502.s013.docx]

**Text S1. Algorithm to remove eSNPs of high LD from genetics of gene expression datasets.**

Sort eSNPs according to equalized expression P-value

Repeat until sorted list is empty {

Take the first eSNP out from the sorted list

If the eSNP is not in LD with any of the eSNPs

already saved ➔ Save the eSNP

}
